# Astrocytic Ryk signaling coordinates scarring and wound healing after spinal cord injury

**DOI:** 10.1073/pnas.2417400122

**Published:** 2025-04-10

**Authors:** Zhe Shen, Bo Feng, Wei Ling Lim, Timothy Woo, Yanlin Liu, Silvia Vicenzi, Jingyi Wang, Brian K. Kwon, Yimin Zou

**Affiliations:** ^a^Department of Neurobiology, School of Biological Sciences, University of California, San Diego, La Jolla, CA 92093; ^b^Department of Orthopaedics, International Collaboration on Repair Discoveries, University of British Columbia, Vancouver, BC V5Z 1M9, Canada

**Keywords:** spinal cord injury, astrocytes, glial scar, microglia, Ryk

## Abstract

Wound healing after spinal cord injury involves highly coordinated interactions among multiple cell types, which are poorly understood. Astrocytes play a central role in creating a border against the non-neural lesion core. To do so, astrocytes undergo dramatic morphological changes by first thickening and elongating their processes and then overlapping them to form a physical barrier. We show here that the expression of a cell-surface receptor, Ryk, is induced in astrocytes after injury in both rodent and human spinal cords. Astrocyte-specific knockout of *Ryk* dramatically elongated the reactive astrocytes, accelerated the formation of the border, and reduced the size of the scar. Astrocyte-specific knockout of *Ryk* also accelerated the injury responses of multiple cell types. Single-cell transcriptomics analyses revealed a broad range of changes in cell signaling among astrocytes, microglia, fibroblasts, and endothelial cells after astrocyte-specific *Ryk* knockout, suggesting that Ryk not only regulates injury responses of astrocytes but may also regulate signals emanating from astrocytes and coordinate the responses of these cell types. The elongation of astrocyte processes is mediated by NrCAM, a cell adhesion molecule induced by astrocyte-specific conditional knockout of *Ryk* after spinal cord injury. Our findings suggest that Ryk is a promising therapeutic target to accelerate wound healing, promote neuronal survival, and enhance functional recovery.

## Significance

Traumatic spinal cord injury, caused by a primary insult, leads to a much larger secondary injury. Wound healing, essential to stop the secondary injury, involves highly coordinated interactions among multiple cell types. We show here that Ryk expression is induced in astrocytes and inhibits their morphological changes by inhibiting the expression of Neuronal Cell Adhesion Molecule (NrCAM). Astrocytic Ryk signaling regulates not only the injury responses of astrocytes but also signals from astrocytes to other cell types, including microglia, fibroblasts and endothelial cells. In spinal cord injury patients, Ryk is induced in both astrocytes and the injured axons. Therefore, Ryk is a promising therapeutic target to accelerate wound healing, promote neuronal survival and connectivity and enhance functional recovery.

Traumatic spinal cord injury starts with a primary injury to neurons and glia and continues with a much larger secondary injury caused by inflammation, ischemia, excitotoxicity, and oxidative stress, which ends with the formation of a fibrotic scar bordered by astrocytes (1). Injured axons initially retract from the lesion site due to the repulsive function of the reinduced guidance cues, such as the Wnt family proteins (2–5). Regrowth of axons or axonal branches toward the injury site is limited, and regrowing axons or axonal branches typically cannot cross the fibrotic scar (6). However, injured axons and their collateral branches can grow around the injury site and extend beyond the injury site through the spared tissue, bypassing the lesion site. Collateral branches of injured axons can also grow from intact axon segments away from the injury site. Uninjured axons can also grow new sprouts (7). This axonal growth, shortly after injury, may lead to new synaptic connections and spontaneous functional recovery in humans and experimental animals. Therefore, increasing the protection of the spared spinal cord tissue and promoting the regenerative growth of axons or axonal branches can enhance the repair of circuits and recovery of sensorimotor functions after spinal cord injury. The understanding of the role of astrocytes after spinal cord injury has been evolving, and more recent studies highlight the benefit of the astrocytes in forming a protective border (8, 9). Reactive astrocytes undergo extensive morphological changes after injury, including initial thickening and then polarization/elongation and finally overlapping of their processes to establish the border (10). The signaling mechanisms regulating these morphological changes are not well known. In addition, astrocytes have complex roles in the inflammatory responses, both proinflammatory and anti-inflammatory, likely temporally and spatially controlled. How these processes are controlled in the astrocytes is not well understood (11). Finally, astrocytes are known to communicate with many other cell types through extensive cell–cell signaling (11). What are these signals and how do these communications affect wound healing are not clear. Here, we report that Ryk, a receptor in both canonical and noncanonical Wnt signaling pathways, plays central roles in regulating astrocyte morphology, differentiation, and communication with multiple cell types for wound healing after spinal cord injury.

## Results

### Ryk Expression Is Induced in Injured Spinal Cords in Mice and Humans.

**Fig. 1.**
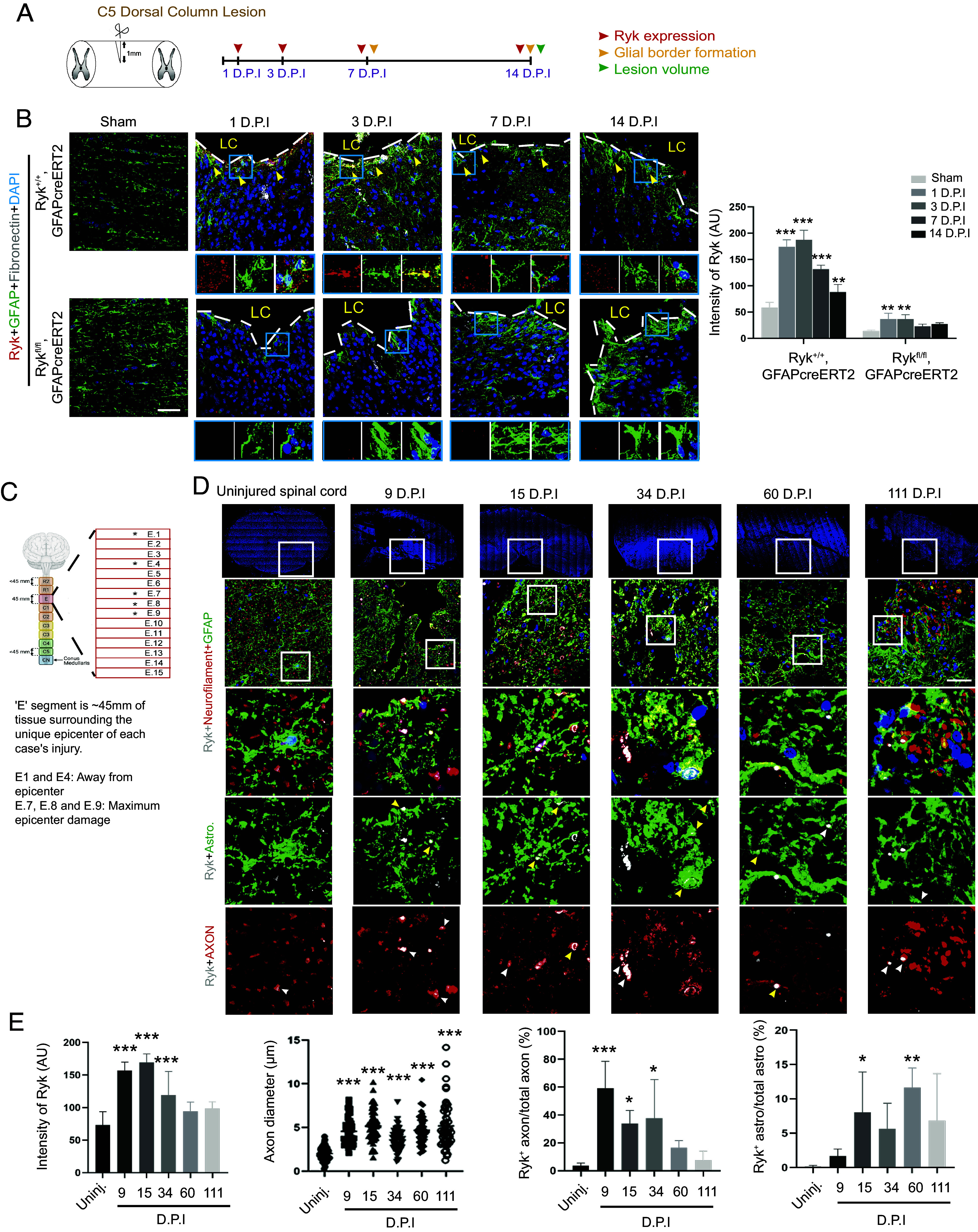
Induction of Ryk expression at injury sites in mice and humans after spinal cord injury. (*A*) Experimental design. Red, orange, and green arrowheads indicate the time of the experiments to test Ryk expression, glial border formation and lesion volume, respectively. (*B*) Immunohistochemistry with antibodies against Ryk, GFAP, and fibronectin 1, 3, 7, or 14 d after C5 dorsal column lesion in control or in *Ryk^fl/fl^* crossed with *GFAPcre^ERT2^*. N = 3 for each group. (Scale bar, 40 µm.) (*C*) Schematic showing the location of the human spinal cord tissue sections relative to the lesion epicenter. (*D*) Immunohistochemistry at different time points after human spinal cord injury with antibodies against Ryk (gray), Neurofilament H (red), and GFAP (green). (*E*) Bar graphs showing quantification of Ryk expression levels, axon diameter, ratio of Ryk^+^ axons to total axons, and ratio of Ryk^+^ astrocytes to total astrocytes. (Scale bar, 40 µm.) Data are expressed as mean ± SD. *P < 0.05, **P < 0.01, and ***P < 0.001 vs. the indicated groups.

The expression of a Wnt receptor, Ryk, known to mediate axon repulsion in development as well as in injured adult spinal cord, was also observed induced in the lesion area in addition to axons, particularly on the border of the developing scar, after spinal cord injury (2, 5, 12). We therefore analyzed Ryk expression and asked whether it is expressed in reactive or border-forming astrocytes. To validate Ryk staining and astrocyte identity, we crossed *Ryk* floxed allele with *hGFAPcre^ERT2^* or *Aldh1L1 cre^ERT2^* and performed C5 dorsal column lesion (5). Mice were subjected to cervical 5 (C5) dorsal column lesion and costained with a polyclonal Ryk antibody previously made in the lab and an astrocyte marker, GFAP, or a fibroblast marker, fibronectin, 1 d, 3 d, 7 d, and 14 d after injury (Fig. 1*A*) (2, 13). Studies reported that astrocyte density was significantly increased within 250 µm from the lesion border (10, 14). Therefore, we examined Ryk expression in those areas and we found that Ryk was significantly increased 1 d, 3 d, and 7 d after spinal cord injury and started to decrease 14 d after injury (Fig. 1*B*). And the Ryk immunoreactivity was found colocalized with astrocyte marker, GFAP (Fig. 1*B*, blue insets). Compared with WT animals, we found that most of the Ryk immunoreactivity was abolished in astrocytes in *Ryk cKO* mice (Fig. 1*B*), confirming the specificity of the Ryk antibody and the effectiveness of the *Ryk* conditional knockout in astrocytes.

To test the relevance of Ryk function in human spinal cord injury, we analyzed spinal cord samples from 5 different deidentified patients at different time points of injury. From each patient, we analyzed 5 transverse sections, E1, 4, 7, 8, and 9 (*SI Appendix*, Fig. S1*A*). E7, 8, and 9 are the epicenter of the injuries (Fig. 1*C*). E1 is approximately 15 mm away from the lesion center while E4 is approximately 6 mm away from the core. First, we costained with a monoclonal Ryk antibody with an astrocyte marker GFAP and an axon marker Neurofilament H in the lesion core (sections E7, 8, and 9) and found that Ryk (white signal) was significantly increased in human spinal tissues at 9 D.P.I. (C6/7 fracture dislocation/C6 AIS A), 15 D.P.I. (C3/4 hyperextension injury/C4 AIS C) and 34 C.P.I (C6/7 fracture dislocation/C7 AIS A) in both astrocytes and in axons compared with spinal cord tissue from uninjured control (Fig. 1 *D* and *E*). In these transverse sections, the longitudinal axon tracts were cut perpendicular to their trajectories. Ryk is a cell surface receptor and was found localized on the axon membrane after injury. The average diameter of Neurofilament signal was found increased in the lesion core, suggesting axonal swelling after injury. Therefore, Ryk expression is induced on axons at the lesion core shortly after injury and the expression persists for at least 1 mo in the human spinal cord but declines 2 mo after injury (Fig. 1 *D* and *E*). Ryk signal on axons started to decrease at 60 D.P.I. (C4/5 hyperextension injury C4 AIS D) and 111 D.P.I. (C4/5 hyperextension injury C5 AIS A). The expression of Ryk on the human astrocytes, on the other hand, continued to increase and peaked at 2 mo after injury. This is different from mice, where Ryk expression in astrocytes peaks on day 3 postinjury (Fig. 1*B*) whereas Ryk expression on injured axons peaks on 7 d postinjury or later [Fig. 2 of Liu et al. (2)]. We noticed that while some Ryk signals were found on axon membranes only (white arrowheads in red channel), majority of the Ryk signals found on astrocyte processes are also close to or in contact with axons (yellow arrowheads in both channels) (Fig. 1*D* and *SI Appendix*, Fig. S2). Fewer Ryk signals are on astrocyte processes only (white arrowheads in green channel) (Fig. 1*D* and *SI Appendix*, Fig. S2). We also investigated the Ryk expression away from the lesion core with astrocyte marker SOX9 (section E4) (*SI Appendix*, Fig. S3*A*) and axon marker Neurofilament (section E1) (*SI Appendix*, Fig. S3*B*). We found that Ryk expression was also significantly increased 9 d, 15 d, and 34 d after injury and started to decline on day 60 and day 111 in both E1 and E4 sections (*SI Appendix*, Fig. S3*C*). And in E4 and E1 sections, Ryk immunoreactivity colocalized with the astrocyte marker SOX9 and the axonal marker Neurofilament H, respectively.

**Fig. 2.**
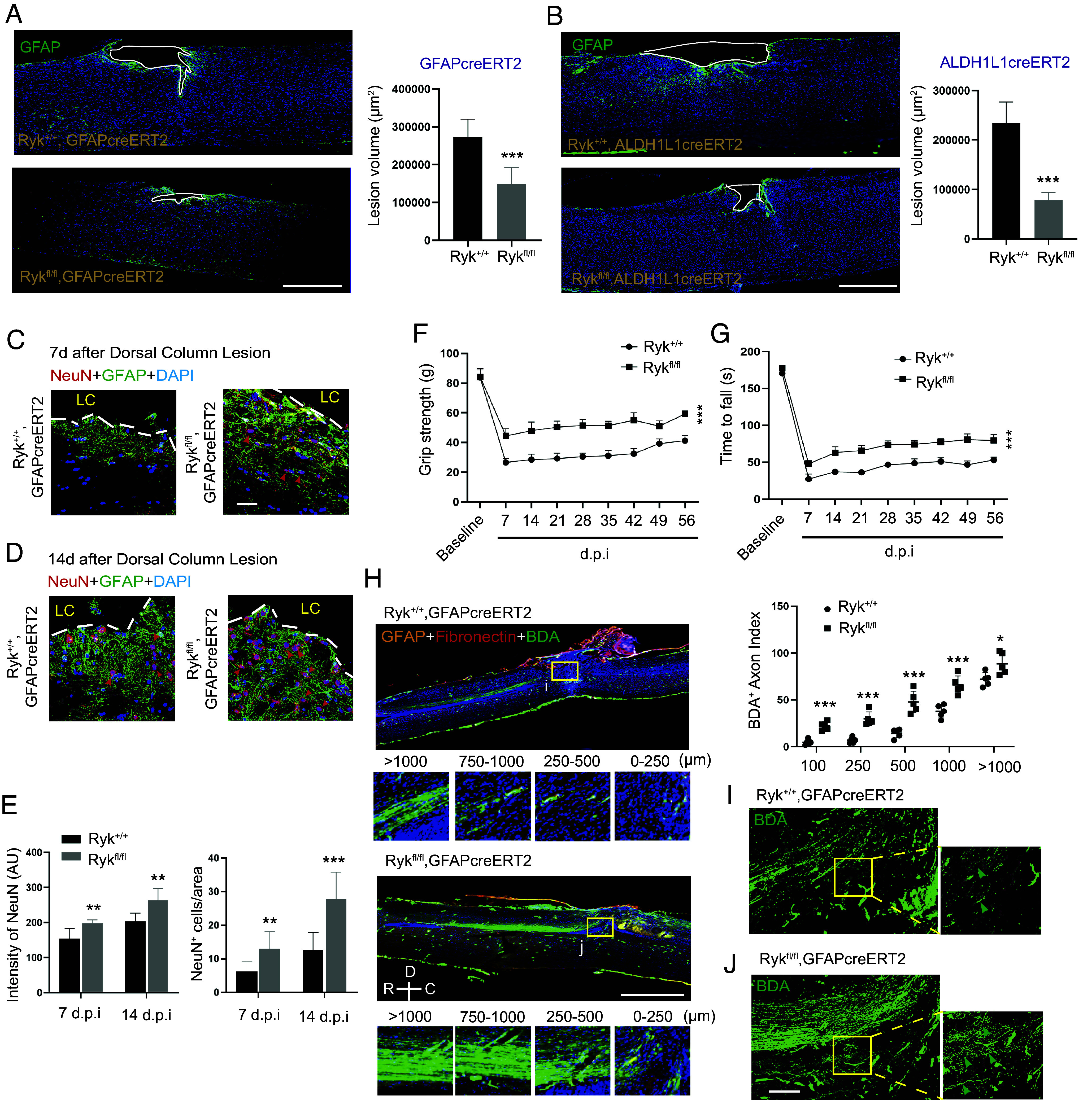
Smaller lesion volume, more preserved neurons and axons and sensory-motor functions in astrocyte-specific Ryk conditional knockout. (*A*) Lesion volume shown in sagittal sections of spinal cord stained with GFAP antibody 14 d after SCI in control or *Ryk^fl/fl^* crossed with *GFAPcre^ERT2^*. (Scale bar, 500 µm.) N = 5 for each group. (*B*) Lesion volume shown in sagittal sections of spinal cord stained with GFAP antibody 14 d after SCI in control or *Ryk^fl/fl^* crossed with *Aldh1L1cre^ERT2^*. (Scale bar, 500 µm.) N = 5 for each group. Immunohistochemistry with NeuN in spinal cord sections 7 d (*C*) or 14 d (*D*) after SCI stained. (Scale bar, 40 µm.) (*E*) Quantification of (*C* and *D*). Measurements of grip strength (*F*) and Rotarod performance (*G*) 2 mo after C5 dorsal column lesion. N = 6 for the control group and n = 7 for *Ryk cKO*. (*H*) Representative images of BDA-labeled CST axons with GFAP and fibronectin staining in control or *Ryk cKO* mice. (Scale bar, 1,000 µm.) N = 5 for each group. Higher magnification of images for control (*I*) or *Ryk cKO* (*J*) from boxed regions (750 µm rostral to the lesion site) indicated in (*H*). (Scale bar, 50 µm.) Data are expressed as mean ± SD. *P < 0.05, **P < 0.01, and ***P < 0.001 vs. the indicated groups.

### Astrocyte-Specific *Ryk* Conditional Knockout Resulted in Smaller Lesion Volume, More Preserved Neurons and Axons and Sensory-Motor Functions.

To explore the role of the induced Ryk in astrocytes, we performed a number of analyses in the injured spinal cords of the astrocyte-specific *Ryk* conditional knockout mice. First, we measured the injury lesion volume based on GFAP delineation in confocal images and observed a significant decrease in lesion volume 14 d after injury in astrocyte-specific *Ryk* knockouts using either *hGFAP-CreERT2* or *Aldh1l1-CreERT2* (Fig. 2 *A* and *B*). We then asked whether the smaller lesion volume promotes the survival of neural tissues and observed that the number and density of NeuN-positive neurons were significantly increased 7 d and 14 d after injury (Fig. 2 *C*–*E*), indicating astrocyte-specific *Ryk cKO* may have promoted neuronal survival. Since astrocyte-specific *Ryk cKO* reduced the lesion size, we further investigated whether there is any long-term functional benefit. Using grip strength and rotarod performance, we found that astrocyte-specific *Ryk cKO* showed more protection of the motor function for at least 2 mo after C5 dorsal column lesion injury (Fig. 2 *F* and *G*, n = 6 for the control group and n = 7 for *Ryk cKO*). And we analyzed the axon morphology 2 mo after injury by injecting biotinylated dextran amine (BDA) into the motor cortex and costained for GFAP and fibronectin. Using a digital stereotactic injector (Item: 51709, Stoelting Co., USA), 0.5 µL of biotinylated dextran amine (BDA; MW 10,000; 10% in PBS; Molecular Probes) was injected into one of the 10 total sites (5 sites/side). We found that *Ryk cKO* significantly reduced the corticospinal tract axon retraction (Fig. 2*H*, normalized to pyramidal tract labeling in the brainstem) and increased the axon branch (Fig. 2 *I* and *J*). Therefore, astrocyte-specific *Ryk cKO* shows long-term benefit.

### Astrocyte-Specific *Ryk* Conditional Knockout Promoted Polarization and Elongation of Astrocyte Processes and Accelerated Astrocyte Border Formation.

**Fig. 3.**
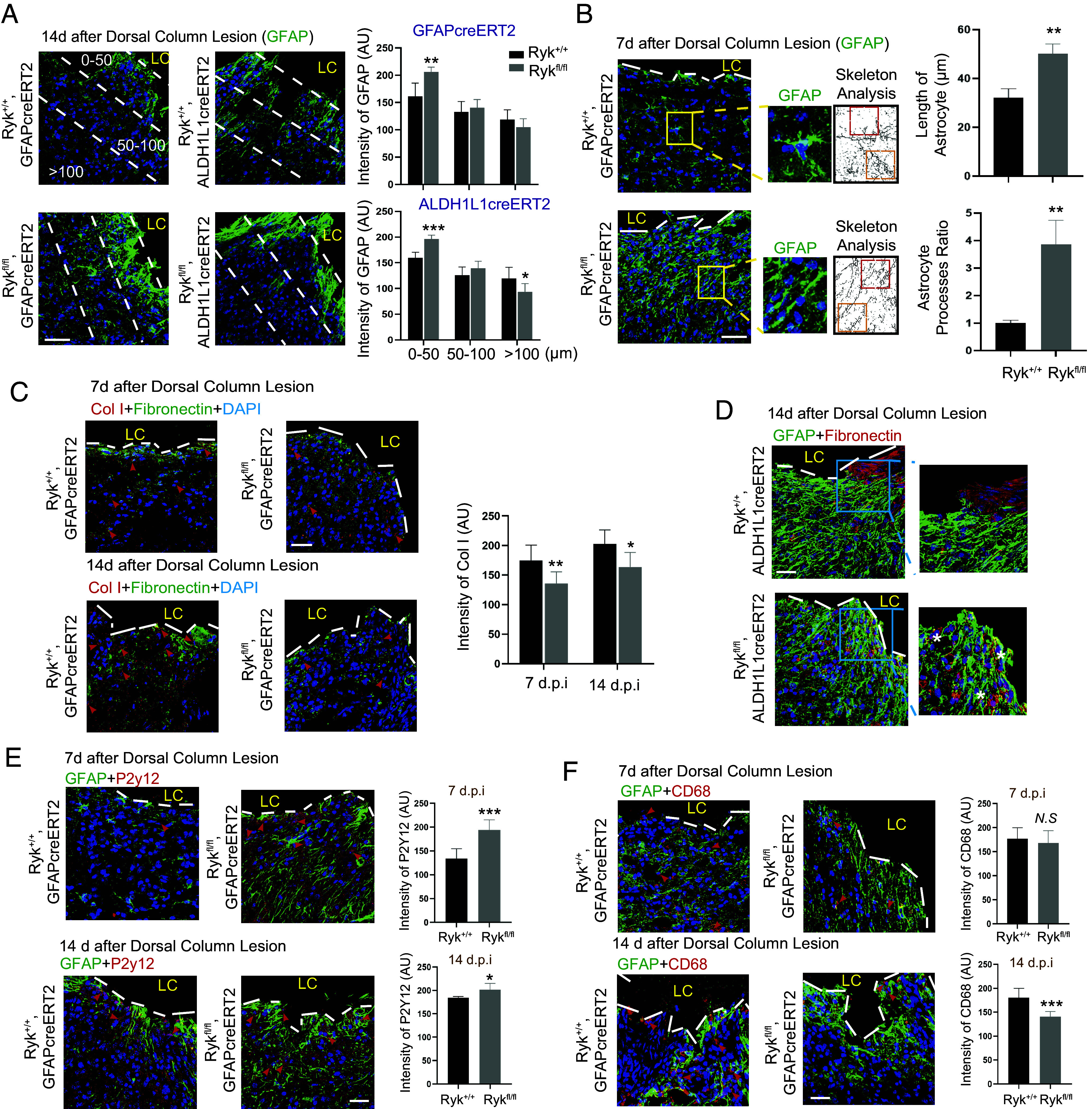
Accelerated astrocyte border formation, enhanced polarization and elongation of astrocyte processes, reduced fibrosis and accelerated inflammatory responses of microglia in astrocyte-specific *Ryk* conditional knockout. (*A*) Astrocyte border strained with GFAP antibody 14 d after SCI in control or *Ryk cKO.* (Scale bar, 40 µm.) Bar graphs show GFAP staining at different distances from the lesion border. N = 5 for each group. (*B*) Skeleton analysis for astrocytes around the lesion border 7 d after SCI. Astrocyte processes ratio: Length of processes toward the lesion site/Length of processes on the other side. (Scale bar, 40 µm.) N = 5 for each group. (*C*) Staining of spinal cord sections with antibodies against Col1 or fibronectin 7 d or 14 d after SCI. (Scale bar, 40 µm.) Bar graphs showed quantification of Col I expression. (*D*) Staining of spinal cord sections with antibodies against GFAP and fibronectin 14 d after SCI. The GFAP^+^ elongated astrocyte formed ovoid-like structures surrounding fibroblasts (* labeling). (Scale bar, 40 µm.) (*E*) Staining of spinal cord sections with antibodies against GFAP and P2Y12 7 d or 14 d after C5 dorsal column lesion in control or *Ryk cKO.* (Scale bar, 40 µm.) N = 3 for each group. (*F*) Staining of spinal cord sections with antibodies against GFAP and CD68 7 d or 14 d after C5 dorsal column lesion in control or *Ryk cKO*. (Scale bar, 40 µm.) N = 3 for each group. Data are expressed as mean ± SD. *P < 0.05, **P < 0.01, and ***P < 0.001 vs. the indicated groups.

To better understand the role of Ryk in astrocytes and how astrocyte-specific *Ryk* knockout affects injury responses, we analyzed the astrocyte border. In addition to the smaller lesion size, we found that the astrocytes form the astrocyte border more efficiently in astrocyte-specific *Ryk cKOs* 14 d after injury (Fig. 3*A*), suggesting that astrocyte-specific *Ryk cKO* accelerated the injury response of astrocytes. In support of this, we found that astrocyte processes were greatly elongated and highly polarized toward the injury site in *Ryk cKO*, particularly obvious 7 d after SCI when the density of GFAP-positive reactive astrocytes is still sparse and when it is possible to clearly observe their morphology (Fig. 3*B* and *SI Appendix*, Fig. S4, mainly quantified in areas with sparse GFAP staining). These highly polarized astrocyte cell processes were also more overlapped in *Ryk cKO* (Fig. 3*B* and *SI Appendix*, Fig. S4). Overlapping of processes is one of the key morphological features of border-forming astrocytes. To further characterize the changes of astrocyte response, we stained for an astrocyte marker, SOX9, and found that the number of SOX9-positive astrocytes was significantly increased 7 d after SCI in *Ryk cKO* (*SI Appendix*, Fig. S5 *A* and *B*), indicating *Ryk cKO* accelerated the process of reactive astrogliosis. The increase 14 d after SCI is much less than at day 7. Furthermore, to test whether the change of the astrocyte border in *Ryk cKO* was caused by astrocyte proliferation, mice were administered a single injection of BrdU on each day from 2 to 7 d after SCI. And we found that the newly proliferated astrocyte significantly increased on day 7 after injury (*SI Appendix*, Fig. S5*C*). However, there was no significant difference between WT and *Ryk cKO* in the number of proliferated astrocytes 14 d after injury, suggesting that we are observing an acceleration of the rate of proliferation shortly after injury rather than a continued increase of astrocyte proliferation (*SI Appendix*, Fig. S5*D*). These data suggested that *Ryk cKO* accelerated the proliferation of reactive astrocytes after injury to form the border, which was observed on day 7. Once enough astrocytes were generated, proliferation slowed down.

### Increased Carrolling of Fibroblasts by Astrocytes Close to the Lesion Border and Accelerated Resolution of Inflammatory Responses of Microglia in Astrocyte-Specific *Ryk* Conditional Knockout.

In addition to the changes of astrocyte morphology at the lesion border in *Ryk cKO*, we also observed changes in the behavior of fibroblasts at the lesion border. We costained for Col I and fibronectin and found that astrocyte-specific *Ryk cKO* reduced immunoreactivity of Col I in the spinal cord tissue close to the lesion border 7 d and 14 d after injury, whereas the level of fibronectin was unchanged (Fig. 3*C* and *SI Appendix*, Fig. S6 *A* and *B*). Fourteen days after injury, astrocytes formed a distinct boundary between the lesion core and the parenchyma in both WT and *Ryk cKO* (Fig. 3*C*). In the WT lesion core, we observed aggregated fibroblasts with strong fibronectin expression. However, in *Ryk cKO*, we found that the fibronectin staining was greatly reduced in the lesion core. On the contrary, fibronectin^+^ cells became enriched in the parenchyma (marked with stars) and appear surrounded by the elongated astrocyte processes (Fig. 3*D*). These results are consistent with the reported observation of carrolling of fibroblasts by astrocytes (10).

P2y12^+^ microglia are homeostatic microglia. The immunostaining data showed that P2y12 expression in *Ryk GFAPcre ERT2 mice* was higher compared to WT mice both 7 d and 14 d after injury (Fig. 3*E*). And in *Ryk Aldh1l1creERT2* mice, P2y12 was also found significantly increased 7 d after injury while there was no significant difference 14 d after injury (*SI Appendix*, Fig. S7*A*). These results suggest that there was an enhanced or accelerated microglia responses in astrocyte-specific *Ryk cKO*. CD68^+^ microglia are phagocytic. Our results showed that CD68 expression did not show significant difference 7 d after injury while a significant decrease in *Ryk cKO* 14 d after SCI (Fig. 3*F* and *SI Appendix*, Fig. S7*B*). These data suggest that inflammation progressed faster and was resolved earlier in astrocyte-specific *Ryk* cKO.

### Single-Cell Transcriptomic Analyses Predicted Extensive Changes of Cell–Cell Signaling in Astrocyte-Specific *Ryk* Knockout.

**Fig. 4.**
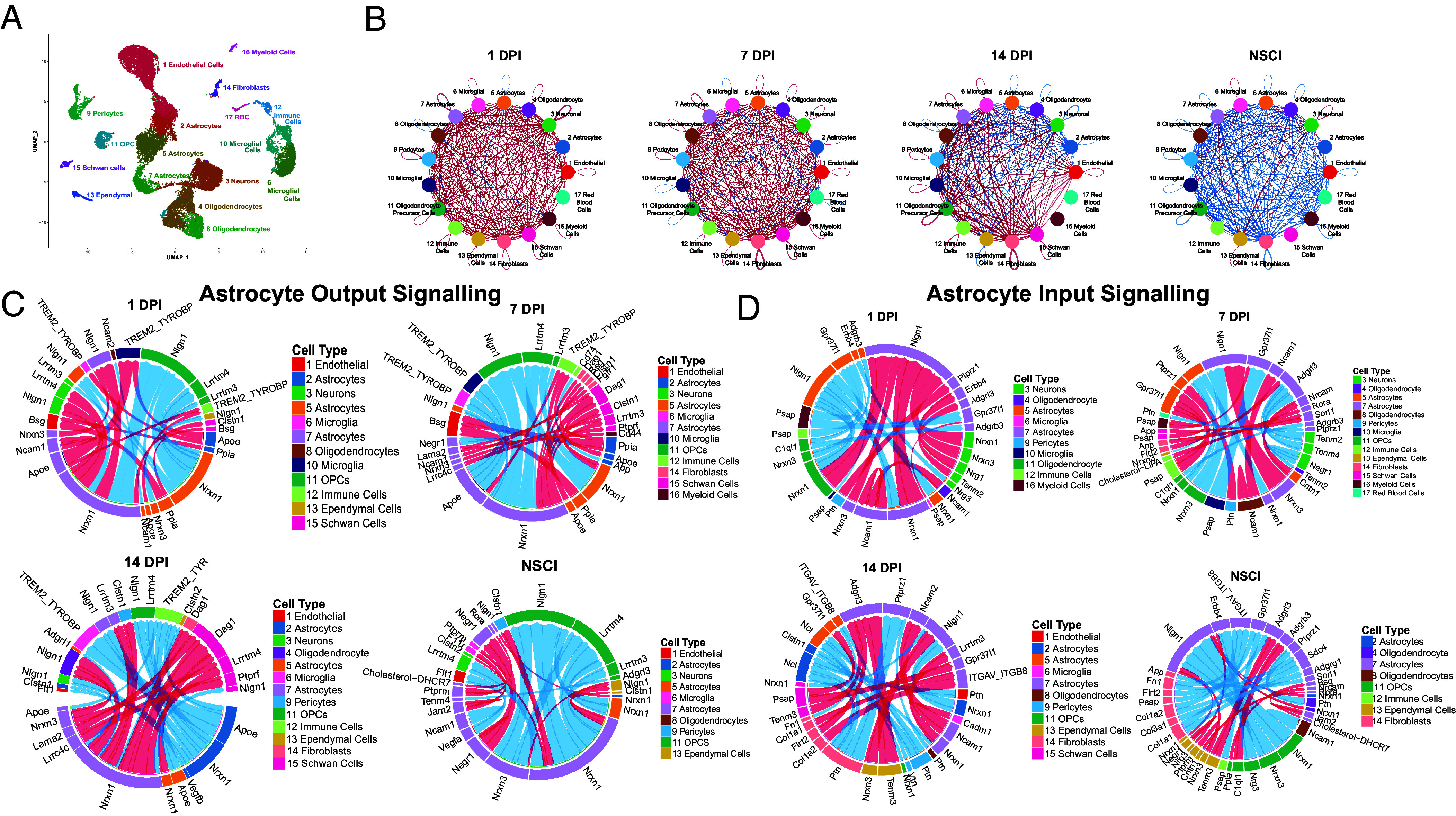
Predicted changes of cell–cell signaling in astrocyte-specific *Ryk cKO* at the injury site after spinal cord injury. (*A*) UMAP plot of major cell types (18,203 cells). (*B*) CellChat chord diagram showing changes of strength of cell–cell signaling in *Ryk cKO*. Red lines represented increase in signaling strength while blue lines represented decrease. (*C*) The top output signaling changes from astrocytes in *Ryk cKO*. (*D*) The top input signaling changes to astrocytes in *Ryk cKO*.

To explore how astrocyte-specific *Ryk cKO* promoted astrocyte polarization and astrocyte border formation and accelerated inflammatory responses, we performed single-cell RNA sequencing to identify potential changes of gene expression and cell–cell signaling among all cell types. We dissociated cells from the spinal cord tissue around the lesion core. Two biological repetitions of single-cell suspensions were performed from the mice which were uninjured, 1 d, 7 d, and 14 d after SCI with or without *Ryk cKO* by *hGFAP-CreERT2.* The Uninjured controls were euthanized according to the approved procedure by the University of California, San Diego Institutional Animal Care and Use Committee and analyzed 14 d after *Ryk* conditional knockout. Although our data were based on two biological replicates, we found that most of the cells are largely distributed consistently among replicates and each of the 8 experimental conditions has similar numbers of cells in all cell clusters (*SI Appendix*, Fig. S8) data deposited in the NIH GEO database: GSE205029) (15). 17 distinct cell clusters were resolved (Fig. 4*A*). According to the highest differentially-expressed genes and canonical markers used in prior studies, we annotated these 17 clusters as 13 major cell types with distinct expression profiles (Fig. 4*A* and *SI Appendix*, Fig. S9): astrocytes (ASC, containing 3 clusters), microglia (MG, containing 2 clusters), oligodendrocytes (containing 2 clusters), endothelial cells, pericytes, fibroblasts, neuronal cells, ependymal cells, blood cells, and immune cells. We first looked into the astrocyte subtypes. The expression of the reactive astrocyte marker gene GFAP was the highest in Cluster 5 astrocytes (*SI Appendix*, Fig. S10). Other markers Aqp4 (Aquaporin 4) and Gja1 (Connexin43) were highly expressed in Cluster 5 and Cluster 7 astrocytes but not in Cluster 2 astrocytes. And S100B, a marker for more mature gray matter astrocytes, was highly expressed in all astrocytes but more in Cluster 5 and Cluster 2 (16). Known border-forming astrocyte markers, Cdh2, Csgalnact1, and Chst11 were found expressed more in Cluster 7 (*SI Appendix*, Fig. S10) (17). Therefore, Cluster 7 astrocytes are likely border-forming astrocytes. Cluster 2 may be another more differentiated subtype derived from reactive astrocytes (18).

To identify the potential changes of cell–cell signaling, we quantified and visualized the global communication atlas among all cell types using a computational tool, CellChat (19). CellChat predicted that spinal cord injury leads to dynamic changes in a large number of cell–cell signaling pathways over the course of 1 d, 7 d, and 14 d after injury in the wild-type control, especially in fibroblasts, ependymal cells, immune cells, myeloid cells, and microglia (*SI Appendix*, Fig. S11*A*). Signaling events are also predicted to change over the course of 1 d, 7 d, and 14 d after injury in astrocyte-specific *Ryk cKO* (*SI Appendix*, Fig. S11*B*). The number of the non-neuronal cells in the uninjured samples is in general very small, occupying less than 1% of total cells in each condition, but is mostly increased after injury and increased more in astrocyte-specific *Ryk cKO* (*SI Appendix*, Fig. S11*C*). When we directly compared astrocyte-specific Ryk conditional knockout with control, we found that the inferred strengths of interactions among all cell types are in general broadly increased in astrocyte-specific *Ryk cKO* 1 d or 7 d after injury (Fig. 4*B*). The thickness of the red lines indicates the strength of increase. On day 1, largest increases were predicted between neurons and Cluster 7 astrocytes, oligodendrocyte precursor cells, ependymal, as well as between Cluster 5 astrocytes and pericytes and fibroblasts. On day 7, largest increases were predicted between fibroblasts and Cluster 7 astrocytes and pericytes. On day 14, signaling between fibroblasts and oligodendrocyte precursor cells, Cluster 7 astrocytes, Cluster 6 microglia, endothelial cells were still predicted increased, whereas signaling involving Cluster 5 astrocytes, Cluster 2 astrocytes, neurons and other cell types were decreased in astrocyte-specific *Ryk cKO*. The thickness of the blue lines indicates the strength of decrease (Fig. 4*B*). The lists of the pathways which are predicted to undergo increase or decrease due to astrocyte-specific *Ryk cKO* also suggest that astrocyte-specific *Ryk cKO* profoundly may change many signaling pathways (*SI Appendix*, Fig. S12). The largest increases of signaling events in astrocyte-specific *Ryk cKO* were also observed 1 d and 7 d postinjury, whereas decreases of signaling events start to take over 14 d postinjury, suggesting astrocyte-specific *Ryk cKO* sped up the injury responses of multiple cell types (*SI Appendix*, Fig. S11*C*).

To understand how *Ryk cKO* may lead to the dramatic morphological changes of astrocytes, we looked into the cell adhesion pathways. We noticed that the ligand–receptor pair Cntn1-Nrcam was slightly reduced (or unchanged) in astrocyte-specific *Ryk cKO* in uninjured animals (*SI Appendix*, Fig. S12*D*) but was ranked one of the top increased, from Cluster 5 astrocytes to Cluster 7 (border-forming) astrocytes, 7 d after injury (Fig. 4*D* and *SI Appendix*, Fig. S12*B*). This suggests that NrCAM, a known transcriptional target of β-catenin/LEF-1 pathway, may be up regulated by *Ryk cKO* in astrocytes only in injury settings (20). The Psap-GPR37/GPR37L1 neuroprotective and glioprotective signaling was predicted to be among the top increased pathways in Cluster 5 astrocytes, again suggesting that astrocyte-specific *Ryk* knockout promotes neuroprotection and regeneration (21) (Fig. 4*D*). Another important signaling pathway is VGEF, which promotes angiogenesis. We found that VGEF signaling was a top up regulated pathway in astrocyte-specific *Ryk cKO* between astrocytes and endothelial cells and between astrocytes and pericytes (*SI Appendix*, Fig. S13).

### Increased Proregenerative Signaling and Accelerated Astrocyte Differentiation in Astrocyte-Specific *Ryk* Knockout.

**Fig. 5.**
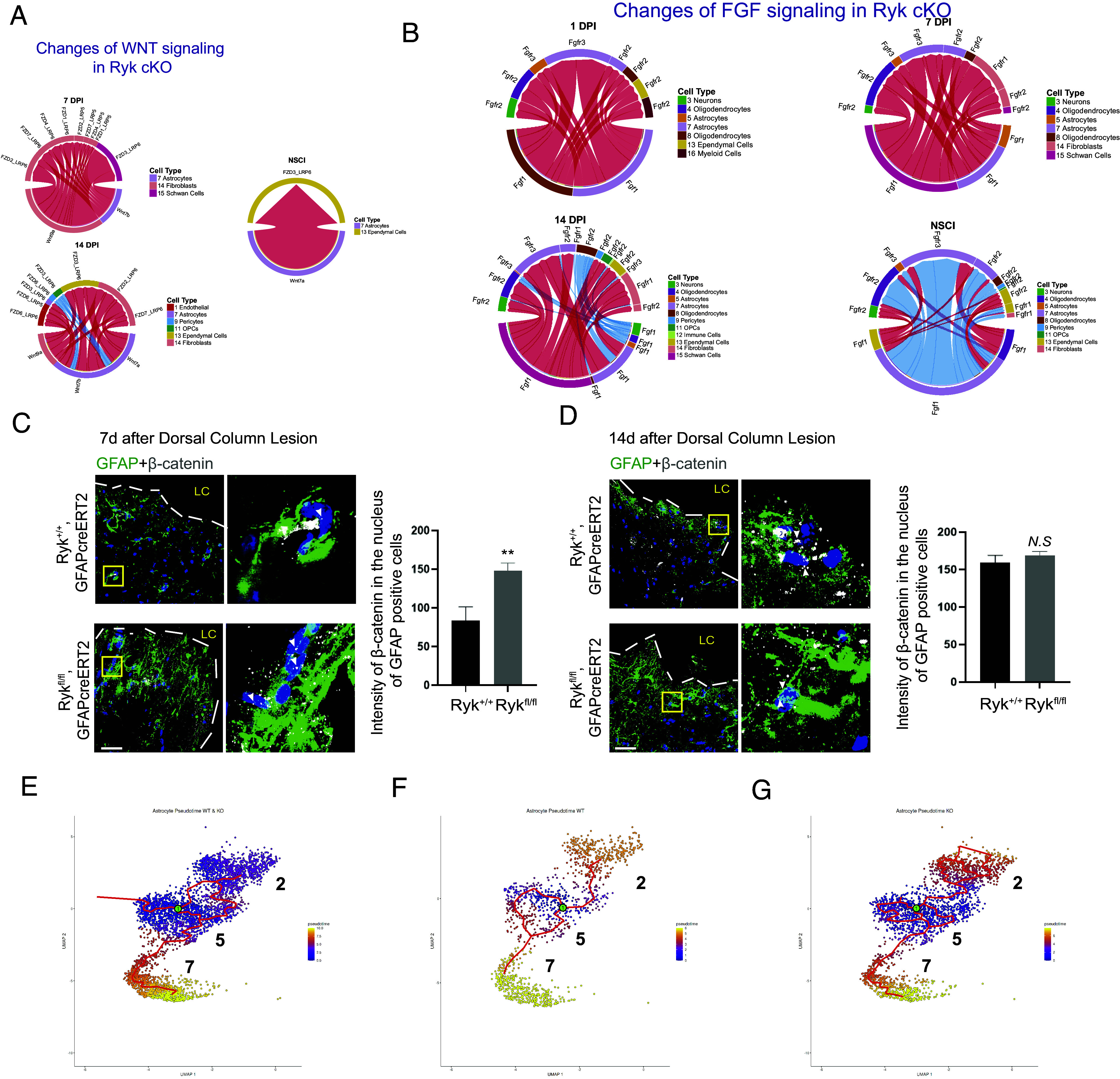
Changes of Wnt and FGF signaling and astrocyte trajectory in astrocyte-specific *Ryk cKO*. (*A*) CellChat analyses predict a strong increase of canonical Wnt signaling involving astrocytes in astrocyte-specific *Ryk cKO*. (*B*) CellChat analyses showing the changes of FGF signaling in astrocyte-specific *Ryk cKO.* (*C*) Immunostaining of spinal cord sections with GFAP and phosphorylated β-catenin 7 d after injury. (Scale bar, 40 µm.) Dot graphs showed quantification of phosphorylated β-catenin levels in astrocyte nuclei. Data are expressed as mean ± SD. ***P < 0.001 vs. the indicated groups. N = 3 animals in each group. (*D*) Immunostaining of spinal cord sections with GFAP and phosphorylated β-catenin 14 d after injury. (Scale bar, 40 µm.) Dot graphs showed quantification of phosphorylated β-catenin levels in astrocyte nuclei. N = 3 animals in each group. (*E*) Pseudotime analyses of astrocyte trajectory in control and *Ryk cKO*. (*F*) Pseudotime analyses of astrocyte trajectory in control. (*G*) Pseudotime analyses of astrocyte trajectory in *Ryk cKO*.

The extensive changes of cell–cell signaling pathways, which are highly relevant to neuroprotection and regeneration prompted us to analyze canonical Wnt signaling, which is essential for activating genes for tissue regeneration and repair after spinal cord injury and can also affect many other signaling pathways (22–25). Ryk is a coreceptor of Frizzled for Wnt and may be a regulator of Wnt signaling in astrocytes after spinal cord injury. CellChat analyses predicted that canonical Wnt signaling pathway was significantly increased from astrocytes to fibroblasts and endothelia cells, as well as from fibroblasts to endothelial cells and fibroblasts to fibroblasts themselves in astrocyte-specific *Ryk cKO* at 7 d and 14 d after injury. Fourteen days after injury, canonical Wnt signaling started to decrease between astrocytes and pericytes and oligodendrocyte precursor cells (Fig. 5*A*). FGF signaling, which regulates glial cell morphology and is also implicated in repair after injury, was significantly increased in astrocytes and between astrocytes and many other cell types after injury in astrocyte-specific *Ryk cKO*, whereas on day 14, FGF signaling between astrocytes and oligodendrocyte precursors, oligodendrocytes started to decrease (Fig. 5*B*) (26–28). To validate the changes of Wnt signaling, we stained for phosphorylated β-catenin and found that it is strongly increased in the nuclei of astrocytes in *Ryk cKO* 7 d after injury (around two-fold) (Fig. 5*C*). Phosphorylated β-catenin levels in control and *Ryk cKO* are similar 14 d after injury, suggesting that the loss of *Ryk* in astrocytes lead to a transient enhancement of canonical Wnt signaling (Fig. 5*D*). Consistent with this, we only observed an increase of BrdU incorporation in astrocytes in *Ryk cKO* 7 d but not 14 d after injury (*SI Appendix*, Fig. S5 *C* and *D*).

The broad changes of cell signaling prompted us to ask more systematically whether the progression of astrocyte differentiation is altered using cell trajectory analysis (29, 30). As GFAP is a marker for reactive astrocytes and S100B (high in type 2) and Cdh2 (border-forming astrocyte marker high in Cluster 7) are markers for more differentiated astrocytes, we selected Cluster 5 as the start point for trajectory analyses (Fig. 5*E* and *SI Appendix*, Fig. S10*B*). *Ryk cKO* significantly promoted the trajectory from Cluster 5 to Cluster 2 and Cluster 7 astrocytes, suggesting accelerated maturation of astrocytes in *Ryk cKO* (Fig. 5 *F* and *G*).

### Single-Cell Transcriptomics Indicated Accelerated Inflammatory Responses and Proregenerative Shift of Microglia in Astrocyte-Specific Conditional *Ryk* Knockout.

**Fig. 6.**
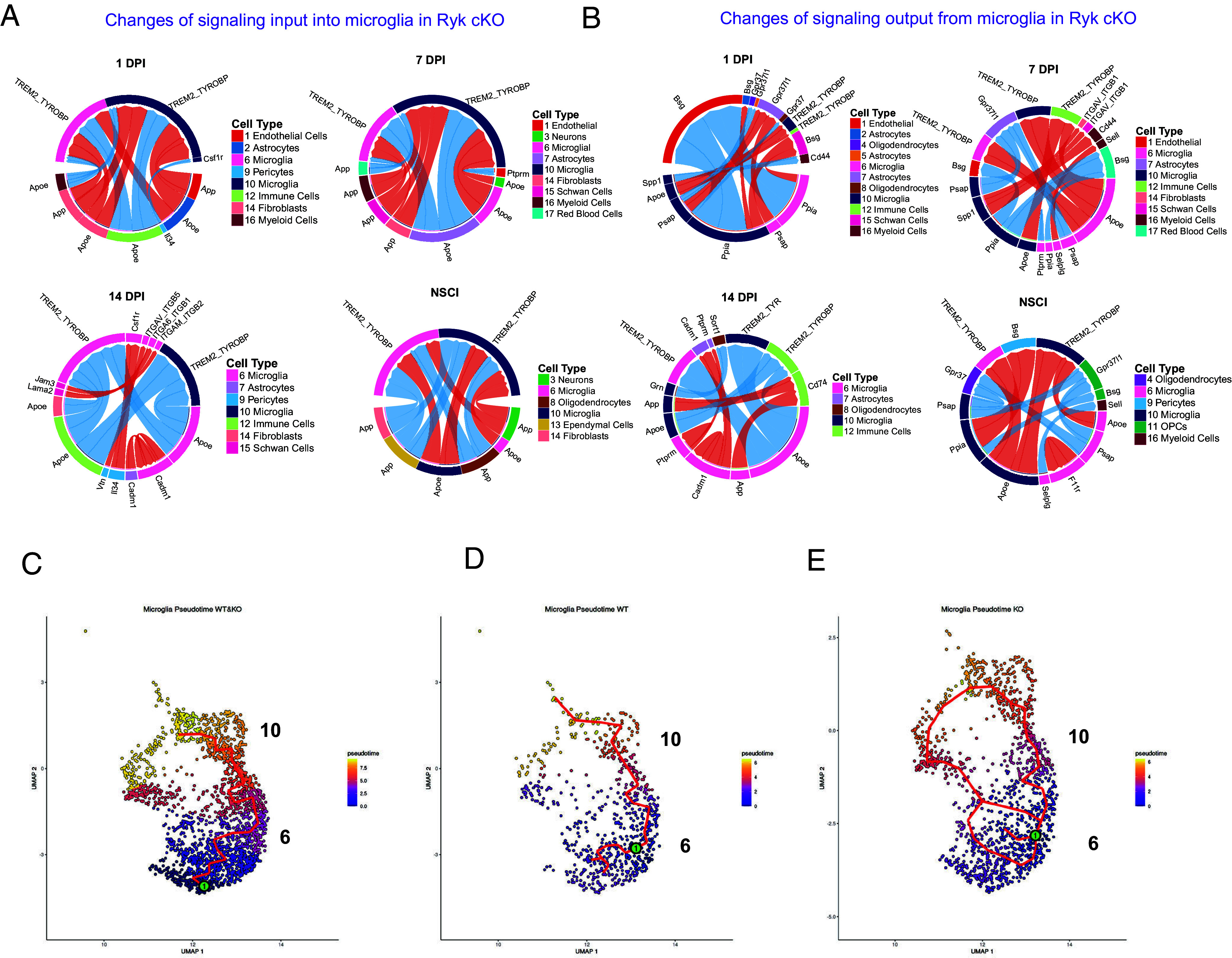
Cell signaling changes in microglia in astrocyte-specific Ryk cKO after SCI. (*A*) The top output signaling changes from microglia in Ryk cKO. (*B*) The top input signaling changes to microglia in *Ryk cKO*. (*C*) Pseudotime analyses of microglia trajectory in control and *Ryk cKO*. (*D*) Pseudotime analyses of microglia trajectory in control. (*E*) Pseudotime analyses of microglia trajectory in *Ryk cKO*.

Microglia also play an important role in astrocyte border and fibrotic scar formation. Studies showed that appropriate microglia activation could remove harmful substances during injury and maintain homeostasis (31, 32). We found that immunoreactivity of P2Y12, a marker for resting microglia, in microglia was increased from 7 d postinjury or earlier but immunoreactivity of CD68, expressed in phagocytic microglia, was reduced 14 d postinjury in astrocyte-specific conditional *Ryk* knockout (Fig. 3 *E* and *F* and *SI Appendix*, Fig. S7) (33–36). The fact that microglia responded to astrocyte-specific conditional *Ryk* knockout at an earlier time point than the formation of the glial border suggests that the accelerated microglia response may not simply be due to reduced tissue damage and the faster closure of the glial border but rather changes of cell–cell signaling. Therefore, we analyzed the responses of microglia in our single-cell sequencing data. CellChat analysis showed that the ApoE-TREM2_TYROBP pathway was a top increased pathway in microglia by on day 1 and day 7. But by day 14, it is decreased, suggesting an earlier activation and progression of inflammatory response (Fig. 6 *A* and *B*). Similar to astrocytes, we performed pseudotime analyses in microglia. We found that Cluster 6 has higher P2Y12 and lower CD68, whereas Cluster 10 microglia have lower expression of P2Y12 but higher CD68 (*SI Appendix*, Fig. S14). Therefore, Cluster 6 may represent the starting point of microglia. We found that astrocyte-specific conditional *Ryk* knockout significantly promoted the trajectory from Cluster 6 to Cluster 10, by increasing the relative proportion of Cluster 10 cells, suggesting accelerated differentiation (Fig. 6 *C*–*E*).

### NrCAM Is Required for the Elongation of the Processes of Reactive Astrocytes and Formation of the Glial Border.

**Fig. 7.**
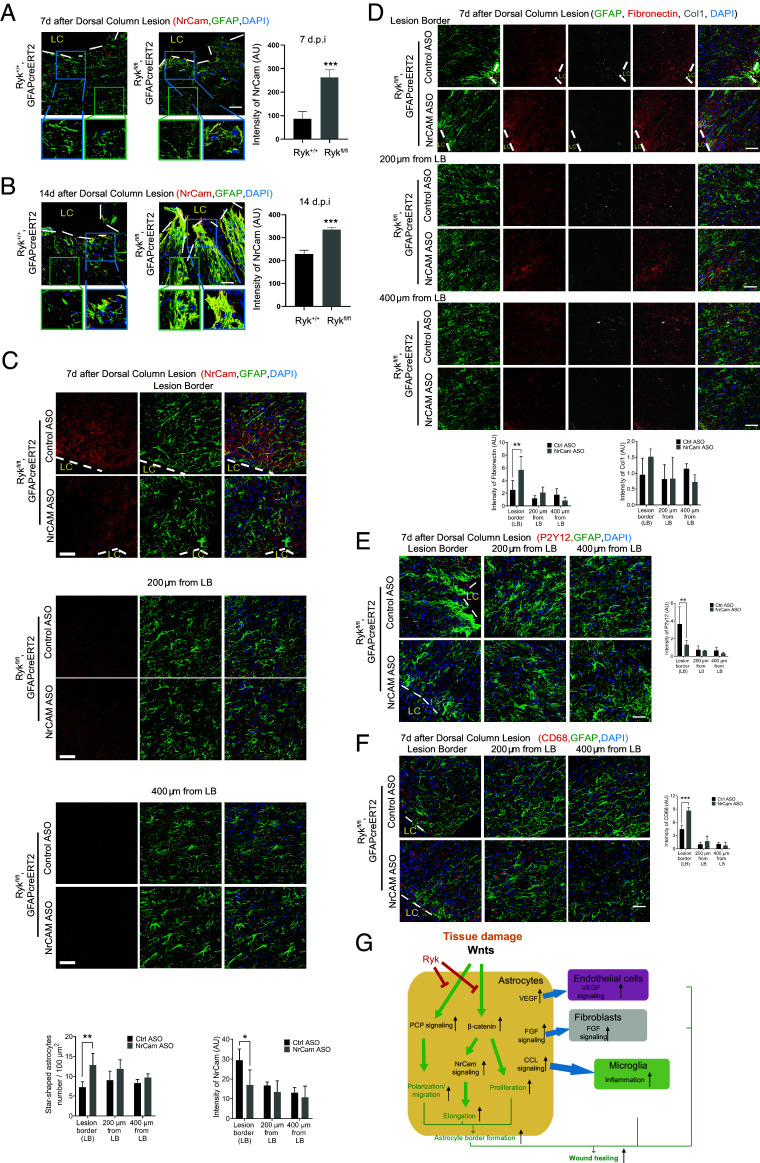
NrCAM is required for the elongation of the processes of border-forming reactive astrocytes. (*A*) Immunostaining of spinal cord sections with antibodies against NrCAM and GFAP in control or *Ryk cKO* 7 d after injury. (Scale bar, 40 µm.) N = 3 for each group. The blue box labeled the areas immediately abutting the lesion core while the green box was about 100 µm away from the lesion core. Bar graphs showed quantitative analysis of the intensity of NrCAM. (*B*) Immunostaining of spinal cord sections with antibodies against NrCAM and GFAP in control or *Ryk cKO* 14 d after injury. (Scale bar, 40 µm.) N = 3 in each group. The blue box labeled the areas immediately next to the lesion core while the green box was about 100 µm away from the lesion core. Bar graphs showed quantification of the intensity of NrCam. (*C*) Immunostaining of spinal cord sections with antibodies against NrCAM and GFAP to analyze the number of star-shaped astrocytes and level of NrCAM in *Ryk cKO* 7 d after C5 dorsal column lesion and ASO injection. N = 3 in each group. (*D*) Immunostaining of spinal cord sections with antibodies against fibronectin, Col1, and GFAP to analyze the level of fibronectin and Col1 in *Ryk cKO* 7 d after C5 dorsal column lesions and ASO injection. (Scale bar, 40 µm.) N = 3 in each group. (*E*) Immunostaining of spinal cord sections with antibodies against P2Y12 and GFAP to analyze the level of P2Y12 in *Ryk cKO* 7 d after C5 dorsal column lesion and ASO injection. (Scale bar, 40 µm.) N = 3 in each group. (*F*) Immunostaining of spinal cord sections with antibodies against CD68 and GFAP to analyze the level of CD68 in *Ryk cKO* 7 d after C5 dorsal column lesion and ASO injection. (Scale bar, 40 µm.) N = 3 in each group. (*G*) Summary of Ryk function in astrocyte border formation, astrocyte differentiation, and cell–cell signaling to coordinate multicellular interactions in wound healing. Data are expressed as mean ± SD. *P < 0.05, **P < 0.01, and ***P < 0.001 vs. the indicated groups.

To test the hypotheses based on the spinal cord injury results and single-cell transcriptomic analyses, we first examined NrCAM expression in astrocytes using immunostaining. We found that there was almost no NrCAM immunoreactivity in astrocytes 7 d after injury in WT mice. However, NrCAM staining was significantly increased in astrocytes in *Ryk cKO* 7 d after injury (Fig. 7*A* and *SI Appendix*, Fig. S15*A*). Fourteen days after SCI, the NrCAM expression level in astrocytes started to increase in the area immediately bordering the lesion core in WT mice. But the NrCAM expression in *Ryk cKO* mice is still much higher than WT (Fig. 7*B* and *SI Appendix*, Fig. S15*B*). These data suggested that *Ryk cKO* might accelerate astrocyte border formation by up-regulating NrCAM signaling in astrocytes.

To further test this, we designed antisense oligos (ASO) against NrCAM to reduce its expression. After confirming the efficiency knock down of NrCAM expression by ASOs by RT PCR, we injected two NrCAM ASOs (separately) into the parenchyma of the spinal cord near the injury site immediately after SCI and obtained similar results (*SI Appendix*, Fig. S16). The immunostaining results verified that NrCAM ASO injection significantly reduced the expression of NrCAM in astrocytes at the lesion border in *Ryk cKO* 7 d after injury but not at 200 μm or 400 μm from the lesion border (Fig. 7*C*). NrCAM ASO reversed *Ryk cKO*-induced astrocyte elongation and polarization 7 d after injury and star-shaped astrocytes, which are rare at the lesion border, were significantly increased at the lesion border (Fig. 7*C*). We then further analyzed fibroblasts and microglia and found that the *Ryk cKO* induced changes at the lesion border (Fig. 3 *C*–*F*) were largely reversed in animals treated with NrCAM ASO (Fig. 7 *D*–*F*). The above data suggested that *Ryk cKO* accelerated astrocyte border formation by up-regulating cell adhesion pathways in astrocytes, which may be critical for the changes of the morphology of the astrocytes. Among them, NrCAM is a major player.

## Discussion

The complex multicellular interactions during wound healing after central nervous system injury are poorly known. Reactive astrocytes play pivotal roles in injury responses in glial border formation. We uncovered a major signaling pathway, which regulates multiple aspects of astrocyte responses (37–39) (Fig. 7*G*). We propose that the canonical Wnt signaling pathway, once activated by injury signals, activates astrocyte proliferation, differentiation (increases of thickness and elongation of astrocyte processes) and inflammatory responses (for tissue debris clearing). Ryk, a Wnt receptor, negatively regulates canonical Wnt pathways in astrocytes in this context. In astrocyte-specific *Ryk* conditional knockout, we found that the expression of a direct transcriptional target of β-catenin, NrCAM, was greatly increased and NrCAM is essential for the elongation of astrocyte processes and potentially migration of astrocytes (20).

How astrocytes coordinate signals to other cell types for wound healing is not well understood. Our study suggests that Ryk may regulate multiple key signals emanating from astrocytes to coordinate responses of many cell types after spinal cord injury (Fig. 7*G*). VEGF, which promotes angiogenesis, is also a direct transcriptional target of the canonical Wnt signaling pathway, and its expression was found increased in astrocyte-specific *Ryk* conditional knockout. The large increase of FGF signaling from astrocytes to fibroblasts in *Ryk cKO* may lead to faster fibroblast proliferation and wound healing, resulting in less inflammation. The accelerated inflammatory responses of both astrocytes and microglia may result in more efficient tissue debris clearing and tissue repair. Because wound healing and tissue repair involve the interactions among many cell types, it is important to coordinate the proliferation and differentiation of all cell types involved. We propose that the normal function of Ryk may be to control the rate of cellular responses to allow time for such coordination. However, it is possible that injury may excessively activate this function of Ryk. Therefore, inhibiting Ryk function may benefit wound healing.

Our study also revealed a potential coordinated morphological polarization of astrocytes and the growth of axons by the same Wnt/Ryk signaling mechanism. Ryk is a Wnt receptor and also regulates a noncanonical Wnt pathway, the planar cell polarity (PCP) pathway, in growth cone guidance and synapse maintenance in neurons (13, 40, 41). Whether the PCP pathway is also active in astrocytes to regulate the polarization of astrocyte processes toward the lesion site deserves future studies. If this is the case, the induced Wnts at the lesion site may prevent astrocyte processes from polarizing toward and/or migrating to the lesion border in similar ways that the axons are repelled away from the injury site (2). Therefore, inhibiting Ryk function in astrocytes can allow astrocyte processes to be better polarized toward and migrate to the lesion border to form the scar border more efficiently to accelerate the glial border formation. And, as we showed previously, the inhibition of Ryk function in axons prevents axon retraction from the lesion site and promote the growth towards the injury site (2–5).

By conditionally knocking out *Ryk* in astrocytes, we were able to accelerate the formation of the glial border and reduce the lesion volume. Our analyses showed that this accelerated astrocyte border formation is beneficial for preserving and recovering sensory-motor functions probably by better protection of the spinal cord tissues due to the faster healing and reduced secondary injury. Our previous work showed that intrathecal infusion of a function-blocking Ryk antibody in the injured spinal cord resulted in greater functional recovery than conditional knocking out *Ryk* from the corticospinal tract (CST) neurons in a skilled motor task (5). Given our findings here, we propose that the additional functional recovery by Ryk antibody treatment may be caused by the faster astrocyte border formation in addition to promoting the growth of CST axons. Therefore, targeting Ryk may have at least two benefits, promoting axon growth and accelerating astroglial border formation to protect spared spinal cord tissue, including neurons. We observed that in injured human spinal cord tissues, Ryk was also induced in both astrocytes and the injured axons, suggesting that Ryk is a promising therapeutic target to treat human spinal cord injury (Fig. 1 *C*–*E*).

## Materials and Methods

All animal work in this research was approved by the University of California, San Diego (UCSD) Institutional Animal Care and Use Committee. Detailed *Materials and Methods*, including surgical procedures, behavioral tests, immunohistochemistry, immunostaining for human spinal cord samples, single-cell RNA seq, and statistical analysis, are included in *SI Appendix*.

## Supplementary Material

Appendix 01 (PDF)

## Data Availability

Single-cell transcriptomics data have been deposited in the NIH GEO database [accession code GSE205029 (Reviewer token: ovylmuaihbgjbcv)] (15). Otherwise, all data are available in the main text or *SI Appendix*.
