# Supplementary material for: Astrocytic Ryk signaling coordinates scarring and wound healing after spinal cord injury
Source: Proc Natl Acad Sci U S A. 2025 Apr 10;122(15):e2417400122. doi: 10.1073/pnas.2417400122 (PMC12012454; doi:10.1073/pnas.2417400122)

MATERIALS AND METHODS

Animals

All animal work in this research was approved by the University of California, San Diego (UCSD) Institutional Animal Care and Use Committee. Animals were housed on a 12 h light/dark cycle and behavioral analyses were done at consistent morning hours during the light cycle. GFAPcreERT2 (Strain #:012849) and Aldh1L1 creERT2 (Strain #:029655) mice were purchased from the Jackson Laboratory. Rykfl/fl (cKO) was generated in the Zou laboratory. Mice aged 8-10 weeks were be used in the following experiments. Mice were induced with Tamoxifen every day for 7 days, starting from 5 days before the operation day. Roughly equal number of mice from both sexes were randomly assigned to experimental groups.

Surgical procedures

C5 dorsal column lesion

Mice were deeply anaesthetized with ketamine, spinal level C5 was exposed by laminectomy and the dorsal columns were lesioned at a depth of 1 mm with Vannas spring scissors (Fine Science Tools, Foster City, CA). The dorsal musculature was sutured with 5-0 silk sutures and the skin was closed with wound clips.

NrCam antisense oligo design and injection

NrCam ASOs are designed and produced by IDT. We designed 3 ASOs against NrCam: NrCam-1 (C*C*A*C*G*C*T*G*A*C*G*C*G*A*A*C*A*T*T*T) NrCam-2 (C*T*G*T*C*G*T*G*C*G*T*G*T*T*T*C*C*G*A*A) and NrCam-3 (G*A*C*G*G*C*T*C*C*T*A*A*T*G*C*G*T*T*T*T). Negative Control ASO: G*C*G*A*C*T*A*T*A*C*G*C*G*C*A*A*T*A*T*G. After efficiency testing by RT-PCR, we used NrCam-2 and -3 for subsequent animal experiments.

ASO injection was performed as described previously. Briefly, immediately after lesion, two injections of ASO were made into the spinal cord along the dorsal midline at 0.5 mm rostral and

0.5 mm caudal to the lesion site. For each injection site, two injections at depths of 0.5 and 1.0 mm were made (0.5 µl at each depth) using a pulled glass capillaries attached to a pneumatic Pico Pump (World Precision Instruments).

Corticospinal Tract Tracing

Corticospinal Tract Tracing was performed as we described previously. Briefly, mice were anesthetized and stabilized in a stereotaxic frame. Using a digital stereotactic injector (Item: 51709, stoelting Co. USA), 0.5 µl of biotin dextran amine (BDA; MW 10,000; 10% in PBS; Molecular Probes) was injected into one of the 10 total sites (5 sites/side). Mediolateral (ML) coordination: 1.5 mm lateral to the bregma; anteroposterior (AP) coordination from the bregma: 1.0, 0.5, 0, 0.75 and 1.5 mm; dorsoventral (DV) coordination: 0.5 mm from the cortical surface. After each injection was completed, the injector tip was left in place for an additional 5 min to ensure that the BDA solution adequately penetrated the tissue. Two weeks later, mice were anesthetized and perfused with 4% paraformaldehyde for detecting CST distribution in the spinal cord.

Behavioral Tests

Grip strength tests

Grip strength was measured with a computerized grip strength meter (Bio-GS3, Bioseb, USA). To measure grip strength in the forepaws of the mice, the experimenter held the mice gently by the base of the tail, allowing the animal to grasp the grid with the forepaws. As soon as the mice grasped the transducer metal grid with their forepaws, the experimenter pulled the animals backwards by the tail until grip was lost. The peak force of each measurement was automatically recorded in grams (g) by the device. Forelimb grip strength in each mouse was measured twice.

Rotarod performance

To measure the balance and ability to coordinate stepping, mice were placed on a single lane rotarod (Med Associates) for two trials per session. The rotarod was set for constant acceleration from 3.0 to 30 rpm over 300 s and mice were scored on seconds to fall. The final result is the average of the two trials.

Immunohistochemistry

Mice were sacrificed and were perfused with saline solution, followed by 4% paraformaldehyde in 0.1 M phosphate-buffered saline. The spinal cords were then dissected out, fixed in 4% paraformaldehyde overnight and equilibrated in 30% sucrose at 4°C. Sagittal sections (30 μm) were cut on a freezing microtome (Thermo, CRYOSTAR NX50). After rinsing with 0.1 % Triton X-100 (vol/vol; Sangon, T0694) in 0.1 M PBS and blocking with blocking buffer (1% bovine serum albumin and 5% donkey serum in TBS solution with 0.1% Triton X-100) for 1 h at room temperature, sections were incubated with the following primary antibodies overnight at 4°C: rabbit anti-Ryk (generated in the Zou laboratory), chicken anti-GFAP (Abcam, ab4674), rabbit anti-GFAP (Abcam, ab7260), mouse anti-GFAP (Sigma, G3893-100UL), mouse anti-fibronectin (Millipore, CP70), rabbit anti-Col 1A1 (Novus Biologicals, NBP1-30054), rabbit anti-NrCam (Abcam, ab24344), rabbit anti-CD68 (Abcam, ab125212),rabbit anti-p2y12 (Invitrogen, PA5-77671) and mouse anti-NeuN (Abcam, ab104224). Afterward, sections were incubated with Alexa Fluor 647- , 594- or Alexa Fluor 488-conjugated secondary fluorescent antibody (1:400; Jackson ImmunoResearch) for 1 h at room temperature, counterstained with 4′,6-diamidino-2-phenylindole (DAPI), and mounted in mounting media. Images were captured using a Zeiss 880 Airyscan microscope.

Immunostaining for human spinal cord samples

These human spinal cord samples, kindly provided by the International Spinal Cord Injury Biobank (ISCIB) located at Vancouver Coastal Health Authority in Canada, were de-identified prior to use in our study. The slides were microwaved slides in Antigen Retrieval Buffer (2 mins at 90% power, 2 mins at 70% power, and 6 mins at 50% power). The slides were then removed from microwave and incubated at room temperature in Antigen Retrieval Buffer for 20 mins and rinsed in 1X TBST at room temperature. Endogenous peroxidase was blocked with 3% H2O2 in 50% methanol for 30 mins. After rinsing with 1X TBST and blocking with blocking buffer for 0.5 h at room temperature, sections were incubated with the following primary antibodies overnight at room temperature: mouse anti-Ryk (5), chicken anti-GFAP (Abcam, ab134436), rabbit anti-Neurofilament (Sigma, N4142) and rabbit anti-SOX9 (Abcam, ab185230). After rinsing with 1X TBST, sections were incubated with Alexa Fluor 647- , 594- or Alexa Fluor 488-

conjugated secondary fluorescent antibody (1:400; Jackson ImmunoResearch) for 1 h at room temperature. After rinsing, slides incubated in 95% ethanol for 1 min at room temperature, in 100% ethanol for 1 min*3 times at room temperature and in xylene for 1 minute*3 times at room temperature. Slides were then incubated with 4′,6-diamidino-2-phenylindole (DAPI) for 15 min, and mounted in mounting media. Images were captured using a Zeiss 880 Airyscan microscope.

Single cell RNA seq

Mice were anesthetized and were performed a trans-cardiac perfusion with fresh ice-cold carbogen-bubbled cutting solution (212 mM sucrose, 1.25 mM NaH_2PO_4, 26 mM $NaHCO_3$, 10 mM Glucose, 3 mM KCl, 7 mM $MgSO_4$, 0.5 mM $CaCl_2$). After decapitation, the spinal cords were immediately extracted and placed in fresh ice-cold cutting solution bubbled with a carbogen gas (95% O_2 and 5% CO_2). And were quickly transferred to the vibratome and sectioned into 300 μm slice. The slices were incubated in ACSF (125 mM NaCl, 1.25 mM NaH_2PO_4, 26 mM $NaHCO_3$, 10 mM Glucose, 2.5 mM KCl, 1.3 mM $MgSO_4$, 2 mM $CaCl_2$) continuously aerated with carbogen gas.

Spinal cords were cut into small pieces < 1 mm in each dimension by a knife and collected to a 60 mm Petri dish leaving only enough ACSF to cover the tissues. The tissues were digested by pronase solution (1 mg/mL pronase, 45 μM ActD, 100 μg/mL DNase I in ice-cold carbogen-bubbled ACSF) at room temperature for 60 min with gentle agitation. After digestion, the tissues were exchanged into trituration buffer (1% FBS, 3 μm ActD, 100 μg/mL DNase I in ice-cold carbogen-bubbled ACSF) and triturated very gently through fire polished salinized Pasteur pipettes with the opening of 600 μm and 300 μm diameter.

The supernatant was collected and centrifuged at 300 rcf for 3 min at 4 °C. The cell pellet was resuspended in wash buffer (0.1% BSA, 3 μm ActD, 100 μg/mL DNase I in ice-cold carbogen-bubbled ACSF) and filtered by 30 μm cell strainer. The dead cells were removed by MACS Dead Cell Removal Kit by manufactory's instruction. The final single cell suspensions were rinsed in cold DPBS solution (0.1% BSA, 3 μm ActD, 100 μg/mL DNase I in ice-cold DPBS). The cell concentration and cell viability were determined by Cell Counter with AOPI staining. In our experiment, the cell viability of all samples was at least 80%. All steps were performed on

ice or at 4 °C except the pronase digestion step. The wide-bore pipette tips were used to resuspend the cell pellet in all steps. Barcoding was performed according to 10X v3.1 feature barcoding protocol (https://www.nature.com/articles/ncomms14049 this is the original v1 barcoding strategy paper). Sequencing was performed in accordance within the 10x protocol on NovaSeq 6000 S4 Paired End

Fastqs were processed by 10X Cell Ranger and aligned to mm10 generated by Cell Ranger mkref. Each barcoded matrix from 10X cell ranger was loaded into R (V 4.1.2) and Seurat (V 4.3.0.1) and read with the Read10X function from Seurat. The percentage of mitochondria genes were used as quality control parameters for each cell. The thresholds used for a high-quality cell was percent mitochondrial genes < 25 and nFeature_RNA > 200. After data quality control, a total of 18,203 high quality cells remained. Batch effects were corrected by Harmony. The cells were then subjected to the standard Seurat pipeline of normalization, feature selection, dimensionality reduction first by principal component analysis (PCA) and then uniform manifold approximation and projection (UMAP). Clustering was computed via Leiden algorithm in Seurat. Seurat's native log normalization was applied to the data and FindVariableFeatures function was used to find top 2000 variable genes used later for the ScaleData function. PCA was run to find 20 PCs which were then corrected using Harmony (V 0.1.1) package accounting for batch and sample effects. Using the corrected PCs from harmony, UMAP was run using Seurat's RunUMAP and clusters were determined by the FindNeighbors and FindClusters function with a resolution parameter of 0.5 and using the Leiden algorithm revealing 17 clusters. To identify the cell-type of the clusters Seurat's FindAllMarkers using default parameters was used (Wilcox ranked sum excluding genes with logFC < 0.25 and percent expression < 0.1). Further, a mixture of logFC and percent expression sorting by each cluster was used to identify marker genes while filtering for significant genes ($p < 0.05$). The top marker genes were compared to known marker genes for each cluster.

All 17 clusters were used for Cell Chat analysis (18). For each experimental group a Cell Chat object was created where default parameters were used for the following Cell Chat functions in this order: identifyOverExpressedGenes, identifyOverExpressedInteractions, computeCommunProb, computeCommunProbPathway, netAnalysis_computeCentrality, and

aggregateNet. To compare differences between cell chat objects, mergeCellChat function was used and rankNet function was used to choose pathways of interest. To visualize pathways a modified netVisual_chord_gene function was used where Down-regulated edges were graphed as blue and upregulated edges were graphed as red in the chord diagram.

Statistical analysis

All data were collected and analyzed in a blind manner. Data are presented as mean ± SD. One-way analysis of variance with least significant difference or Dunnett's T3 post hoc test (where equal variances were not assumed) was applied for multiple comparisons, whereas Student's t-test was used for comparisons between two groups. $P < 0.05$ was considered statistically significant.

Sup Fig 1. Clinical information of spinal cord injury patients whose tissues were analyzed.

Sup Fig 2. Additional examples of Ryk expression on axons and astrocytes in injured human spinal cord.

Sup Fig 3. Induction of Ryk expression in human spinal cord injury away from the lesion epicenter. (A) Immunostaining of human spinal cord tissue (E4) at different time points after injury with antibodies against Ryk and SOX9. **(B)** Immunostaining of human spinal cord tissue (E1) at different time points after injury with antibodies against Ryk and Neurofilament H. **(C)** Bar graphs showing quantifications of Ryk expression level, SOX9 expression level and the percentage of Ryk^+ astrocytes. Data are expressed as mean ± SD. ****P* < 0.001 vs. the indicated groups. Scale bar =40 µm.

Sup Fig 4. Increase of length and polarization of astrocyte processes in astrocyte-specific *Ryk cKO*. (A) Skeleton analysis for astrocytes around the lesion core 7 d after SCI. Scale bar =40 µm. N=5 for each group. ****P* < 0.001 vs. control groups.

Sup Fig 5. Accelerated astrocyte activation and proliferation around the lesion site in astrocyte-specific *Ryk cKO*. (A) Staining of spinal cord sections 7 days and 14 days after SCI with SOX9 antibody in $Ryk^{fl/fl}$ crossed with $Aldh1L1cre^{ERT2.}$ Scale bar =40 µm. Data are expressed as mean ± SD. N=3 for each group. ***P* < 0.01 and ****P* < 0.001 vs. control groups. **(B)** Staining of spinal cord sections 7 days and 14 days after SCI with SOX9 antibody in $Ryk^{fl/fl}$ crossed with $GFAPcre^{ERT2}$**.** Scale bar =40 µm. Data are expressed as mean ± SD. N=3 for each group. ***P* < 0.01 and ****P* < 0.001 vs. control groups. **(C)** BrdU labeling and Ki67 staining in spinal cord sections 7 days after injury. Scale bar =40 µm. N=3 for each group. ***P* < 0.01 vs. control group. **(D)** BrdU labeling and Ki67 staining in spinal cord sections 14 days after injury. Scale bar =40 µm. N=3 for each group. ***P* < 0.01 vs. control group.

Sup Fig 6. Changes of fibroblasts in astrocyte-specific *Ryk cKO*. (**A**) Decrease of immunoreactivity of Col I in astrocyte-specific *Ryk cKO* 7 days after injury. (**B**) Decrease of immunoreactivity of Col I in astrocyte-specific *Ryk cKO* 14 days after injury. Scale bar =40 µm. Data are expressed as mean ± SD. N=3 for each group. **P* < 0.05 and ***P* < 0.05 vs. the indicated groups.

Sup Fig 7. Changes of microglia in astrocyte-specific *Ryk cKO*. (A) Immunostaining of spinal cord sections with antibodies against GFAP and P2Y12 7 days or 14 days after C5 dorsal column lesion in control or astrocyte-specific *Ryk cKO*. **(B)** Immunostaining of spinal cord sections with antibodies against with antibodies against GFAP and CD68 7 days or 14 days after C5 dorsal column lesion in control or astrocyte-specific *Ryk cKO*. Scale bar =40 µm. N=3 for each group. Data are expressed as mean ± SD. ***P* < 0.01 vs. the indicated groups.

Sup Fig 8. Distribution of single cells in individual replicates of single cell sequencing experiments.

Sup Fig 9. Markers for major cell types.

Sup Fig 10. Makers for astrocyte subtypes. (**A**)**.** Dot blots of markers in 3 subtypes of astrocytes. (**B**) Distribution of markers in 3 subtypes astrocytes.

Sup Fig 11. Changes of cell-cell communications after SCI. (A) Cell-cell communication network at different time points in control. **(B)** Cell-cell communication network at different time points in astrocyte-specific *Ryk cKO*. **(C)** Cell counts and percentages of some of the rare non-neural cell clusters in control and astrocyte-specific *Ryk cKO*.

Sup Fig 12. Ranking of signaling pathways which underwent the greatest changes in astrocyte-specific *Ryk cKO*. (**A**) Top pathways in astrocyte-specific *Ryk cKO* without spinal cord injury. (**B**) Top pathways in astrocyte-specific *Ryk cKO* 1 day post-injury. (**C**) Top pathways in astrocyte-specific *Ryk cKO* 7 days post-injury. (**D**) Top pathways in astrocyte-specific *Ryk cKO* 14 days post-injury.

Sup Fig 13. Changes of VGEF signaling in in astrocyte-specific *Ryk cKO.*

Sup Fig 14. Markers for microglia subtypes.

Sup Fig 15. Changes of NrCAM expression in astrocyte-specific *Ryk cKO*. (A) Immunostaining of spinal cord sections with antibodies against NrCam and GFAP in control or *Ryk cKO* 7 days after injury. **(B)** Immunostaining of spinal cord sections with antibodies against

NrCam and GFAP in control or *Ryk cKO* 14 days after injury.. Scale bar =40 µm. Blue box labelled the areas immediately abutting the lesion core while green box was about 100 µm away from the lesion core. Bar graphs showed quantitative analysis of the intensity of NrCam. N=3 for each group. Data are expressed as mean ± SD. ****P* < 0.001 vs. the indicated groups.

Sup Fig 16. Design and validation of NrCAM antisense oligos. (**A**) Sequence of control and 3 ASOs against NrCAM. (**B**) RT-PCR to test the efficacy of antisense oligos.

Sup Fig 1.

ISCIB ID	Age at Injury	Gender	Post-Injury Interval (time between injury & death)	Post-Mortem Interval (time between death and collection)	Mechanism of Injury	Primary Spine Diagnosis - Level (s)	Primary Spine Diagnosis - Description	Neurological level	AIS grade
BB-00001	55	M	9days20hrs53mins	1days0hrs7mins	Transport	C6-C7	Three column fracture dislocation	C6	A
BB-00009	95	M	15days18hrs55mins	1days10hrs5mins	Fall	C3-C4	Hyperextension, avulsion flakes, traumatic disc	C4	C
BB-00004	76	M	[34days]	0days13hrs10mins	Fall	C6-C7	Three column fracture dislocation - closed	C7	A
BB-00006	83	M	[60days]	0days15hrs0mins	Fall	C4-C5	Hyperextension, avulsion flakes, traumatic disc	C4	D
BB-00002	60	M	111days11hrs30mins	5days7hrs35mins	Fall	C4-C5	Hyperextension, avulsion flakes, traumatic disc	C5	A

Sup Fig 2

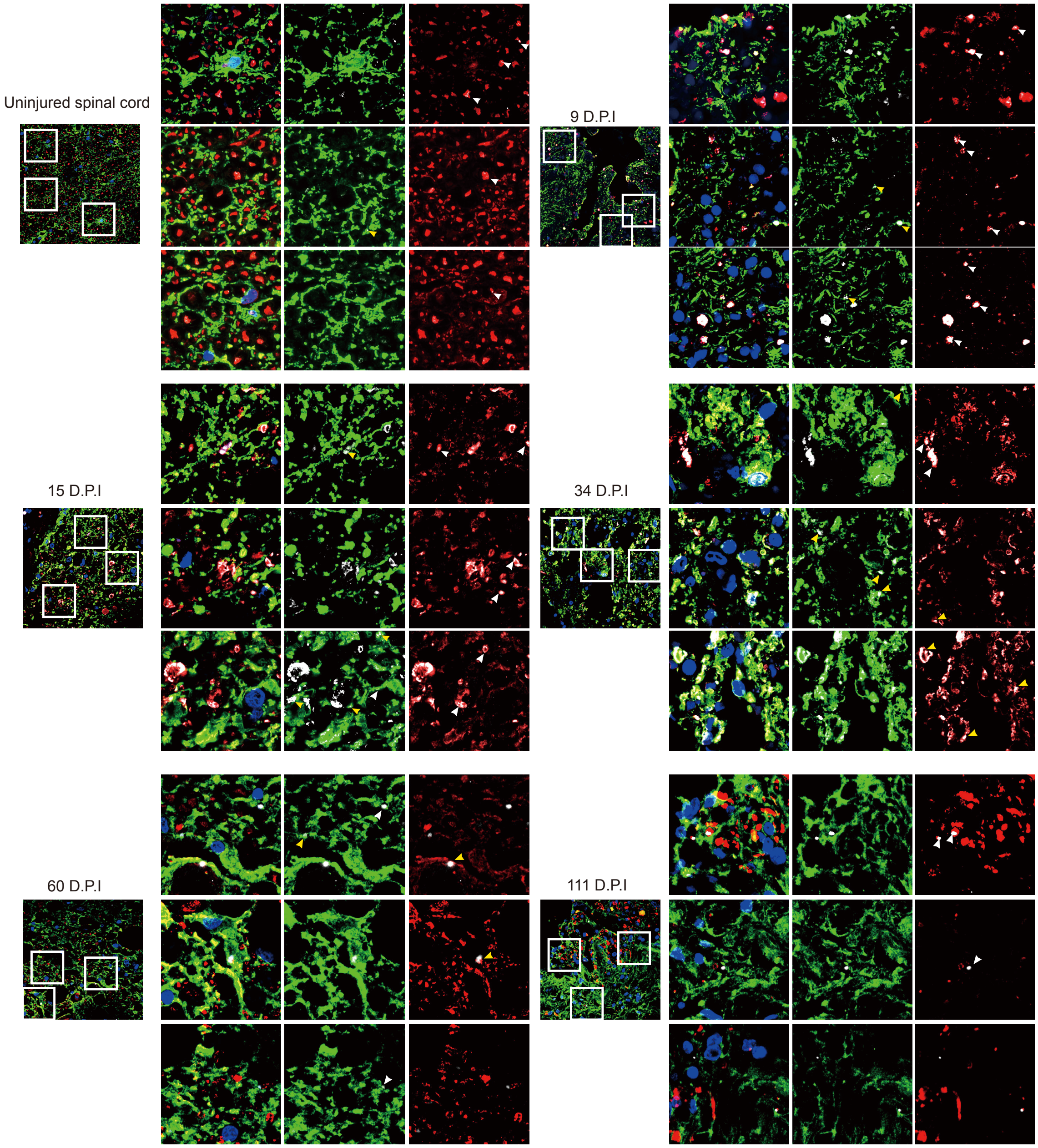

Sup Fig 3.

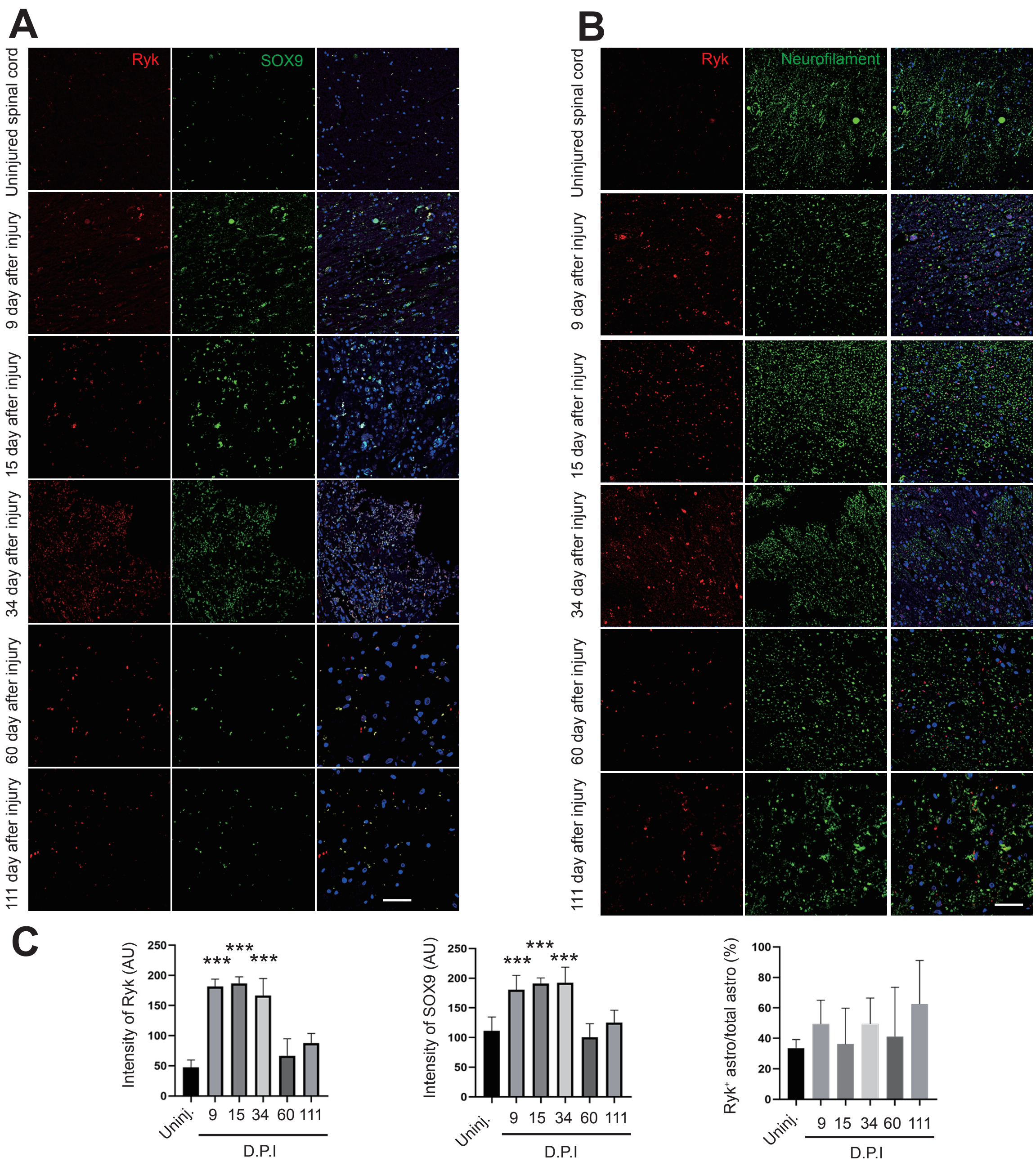
A
Ryk
SOX9
Uninjured spinal cord
9 day after injury
15 day after injury
34 day after injury
60 day after injury
111 day after injury
B
Ryk
Neurofilament
Uninjured spinal cord
9 day after injury
15 day after injury
34 day after injury
60 day after injury
111 day after injury
C
Intensity of Ryk (AU)
Intensity of SOX9 (AU)
Ryk⁺ astro/total astro (%)
Uninj.
9 15 34 60 111
D.P.I
***

Sup Fig 4.

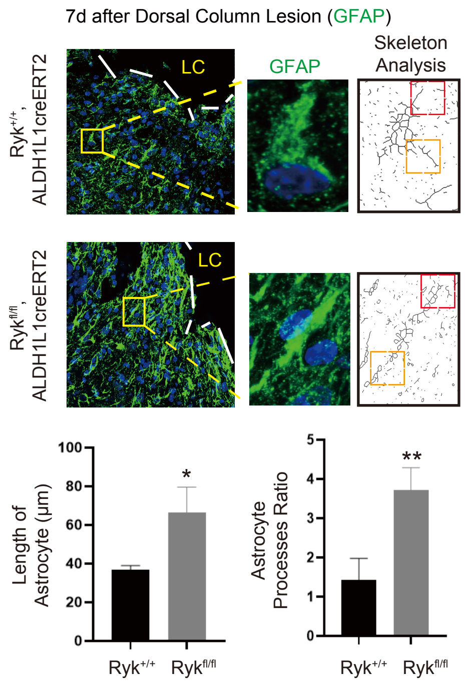

7d after Dorsal Column Lesion (GFAP)
GFAP
Skeleton Analysis
LC
LC
Ryk+/+, ALDH1L1creERT2
Rykfl/fl, ALDH1L1creERT2
Length of Astrocyte (μm)
100
80
60
40
20
0
*
Ryk+/+
Rykfl/fl
Astrocyte Processes Ratio
5
4
3
2
1
0
**
Ryk+/+
Rykfl/fl

Sup Fig 5.

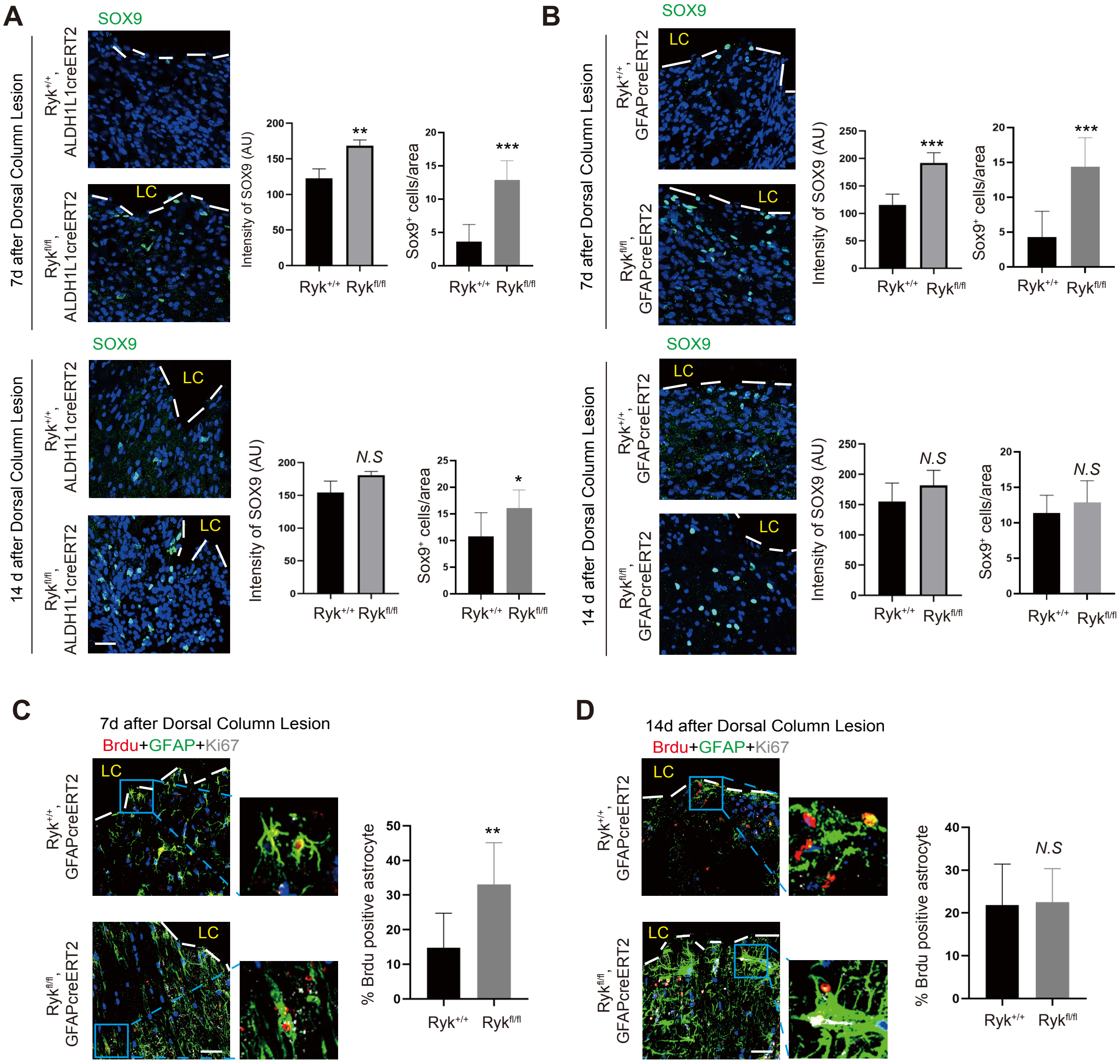
A
B
C
D
SOX9
LC
7d after Dorsal Column Lesion
14 d after Dorsal Column Lesion
Ryk+/+, ALDH1L1creERT2
Rykfl/fl, ALDH1L1creERT2
Ryk+/+, GFAPcreERT2
Rykfl/fl, GFAPcreERT2
Intensity of SOX9 (AU)
Sox9+ cells/area
Ryk+/+
Rykfl/fl
**
***
*
N.S
Brdu+GFAP+Ki67
% Brdu positive astrocyte
7d after Dorsal Column Lesion
14d after Dorsal Column Lesion

Sup Fig 6.

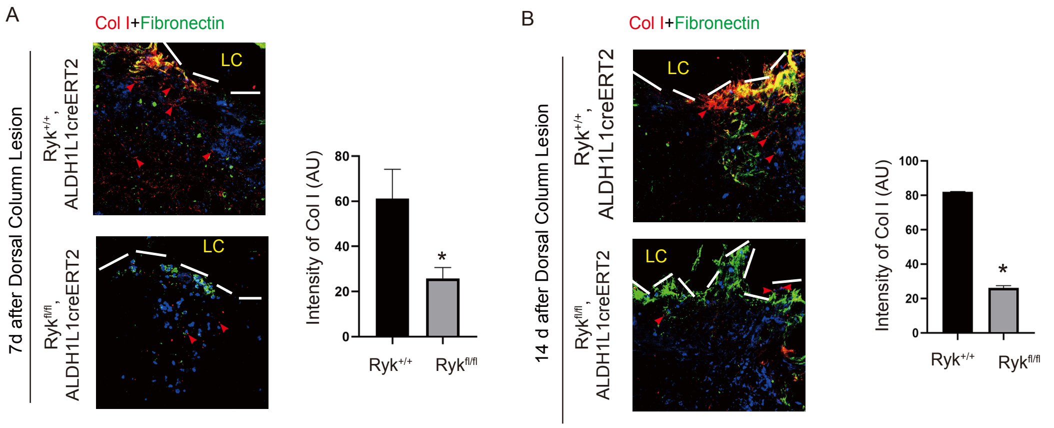
A
Col I+Fibronectin
7d after Dorsal Column Lesion
Ryk+/+, ALDH1L1creERT2
Rykfl/fl, ALDH1L1creERT2
LC
LC
Intensity of Col I (AU)
80
60
40
20
0
*
Ryk+/+
Rykfl/fl
B
Col I+Fibronectin
14 d after Dorsal Column Lesion
Ryk+/+, ALDH1L1creERT2
Rykfl/fl, ALDH1L1creERT2
LC
LC
Intensity of Col I (AU)
100
80
60
40
20
0
*
Ryk+/+
Rykfl/fl

Sup Fig 7.

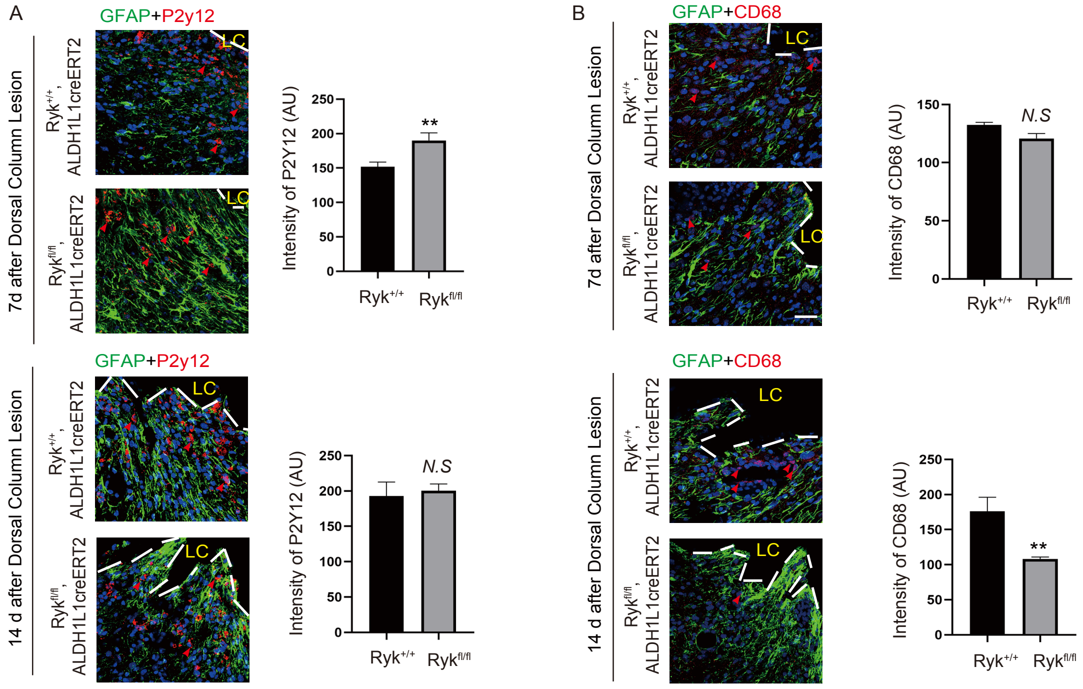
A
GFAP+P2y12
7d after Dorsal Column Lesion
Ryk+/+, ALDH1L1creERT2
Rykfl/fl, ALDH1L1creERT2
LC
Intensity of P2Y12 (AU)
**
Ryk+/+
Rykfl/fl
14 d after Dorsal Column Lesion
N.S
B
GFAP+CD68
Intensity of CD68 (AU)

Sup Fig 8

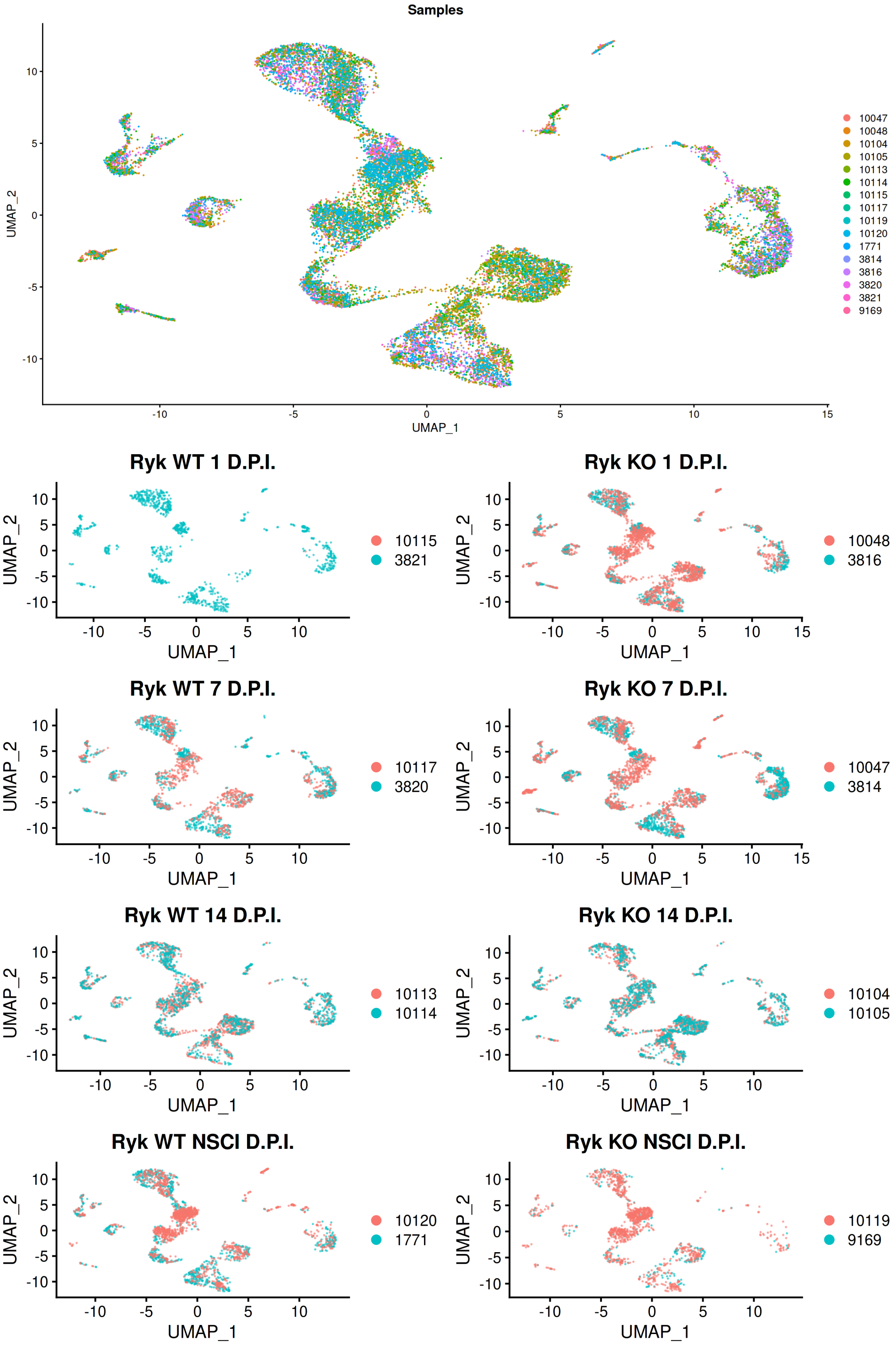
Samples
10047
10048
10104
10105
10113
10114
10115
10117
10119
10120
1771
3814
3816
3820
3821
9169
UMAP_1
UMAP_2
Ryk WT 1 D.P.I.
10115
3821
Ryk KO 1 D.P.I.
10048
3816
Ryk WT 7 D.P.I.
10117
3820
Ryk KO 7 D.P.I.
10047
3814
Ryk WT 14 D.P.I.
10113
10114
Ryk KO 14 D.P.I.
10104
10105
Ryk WT NSCI D.P.I.
10120
1771
Ryk KO NSCI D.P.I.
10119
9169

Sup Fig 9.

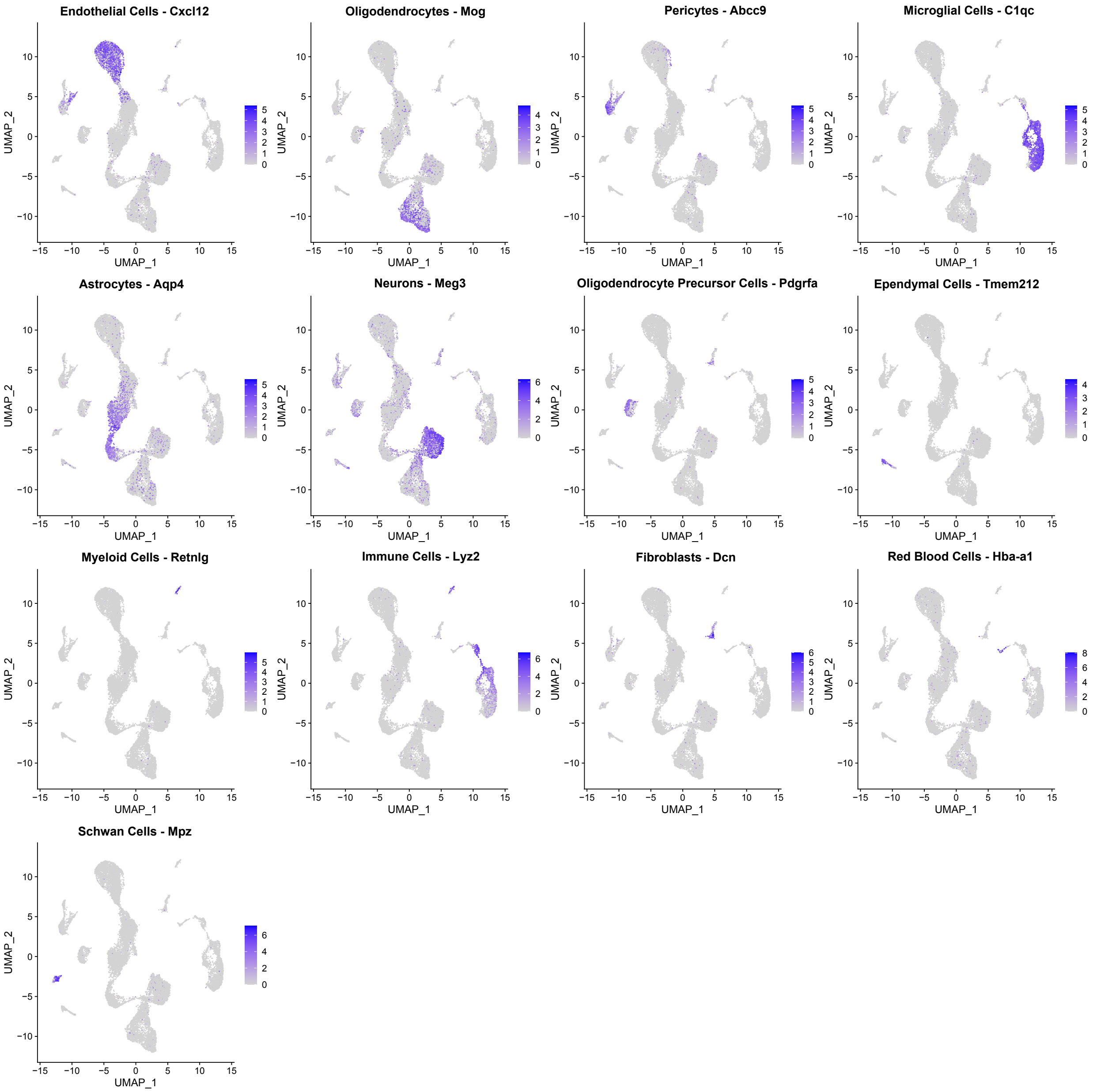
Endothelial Cells - Cxcl12
Oligodendrocytes - Mog
Pericytes - Abcc9
Microglial Cells - C1qc
Astrocytes - Aqp4
Neurons - Meg3
Oligodendrocyte Precursor Cells - Pdgrfa
Ependymal Cells - Tmem212
Myeloid Cells - Retnlg
Immune Cells - Lyz2
Fibroblasts - Dcn
Red Blood Cells - Hba-a1
Schwan Cells - Mpz
UMAP_1
UMAP_2

Sup Fig 10

A

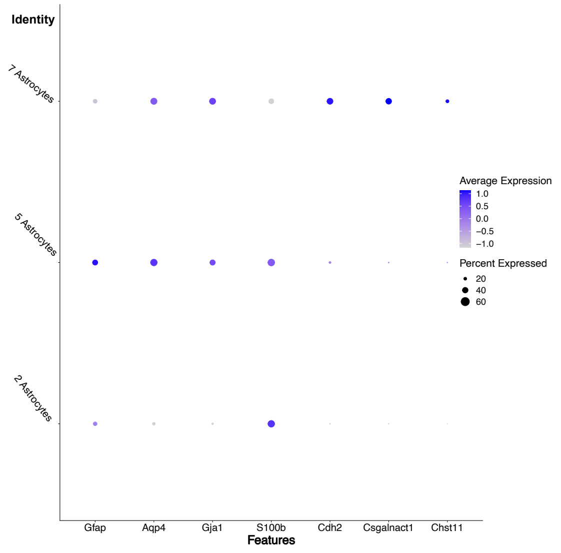
Identity
7 Astrocytes
5 Astrocytes
2 Astrocytes
Average Expression
1.0
0.5
0.0
−0.5
−1.0
Percent Expressed
20
40
60
Gfap
Aqp4
Gja1
S100b
Cdh2
Csgalnact1
Chst11
Features

B

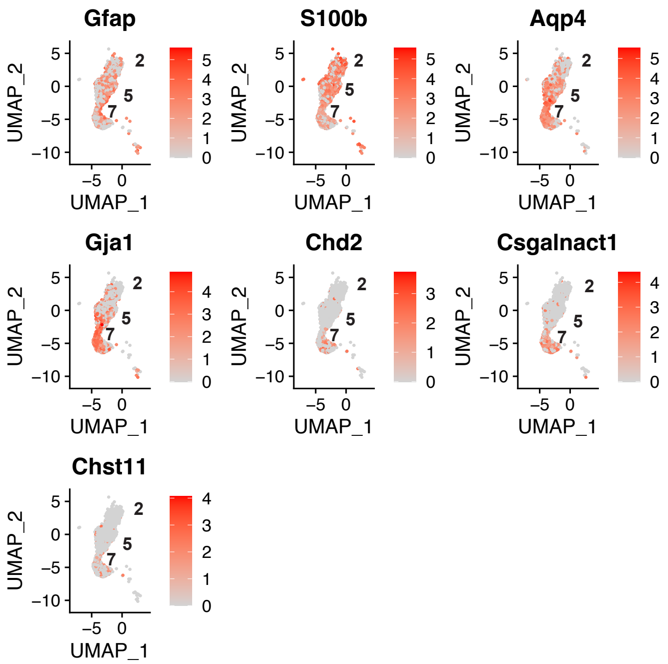
Gfap
S100b
Aqp4
Gja1
Chd2
Csgalnact1
Chst11
UMAP_2
UMAP_1
2
5
7

Sup. Fig. 11

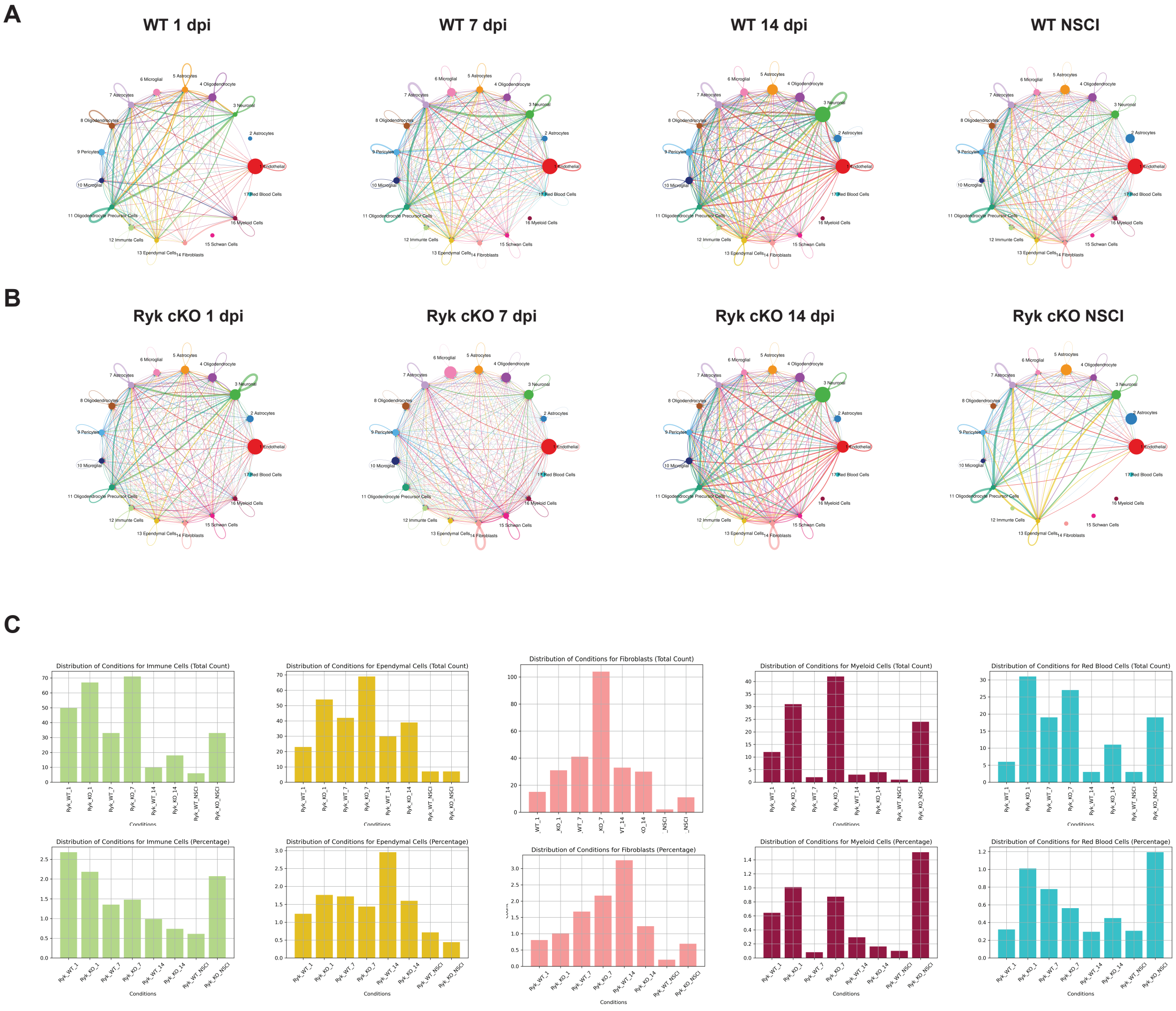
A
WT 1 dpi
WT 7 dpi
WT 14 dpi
WT NSCI
B
Ryk cKO 1 dpi
Ryk cKO 7 dpi
Ryk cKO 14 dpi
Ryk cKO NSCI
C
Distribution of Conditions for Immune Cells (Total Count)
Distribution of Conditions for Ependymal Cells (Total Count)
Distribution of Conditions for Fibroblasts (Total Count)
Distribution of Conditions for Myeloid Cells (Total Count)
Distribution of Conditions for Red Blood Cells (Total Count)
Distribution of Conditions for Immune Cells (Percentage)
Distribution of Conditions for Ependymal Cells (Percentage)
Distribution of Conditions for Fibroblasts (Percentage)
Distribution of Conditions for Myeloid Cells (Percentage)
Distribution of Conditions for Red Blood Cells (Percentage)
Conditions

Sup. Fig. 12

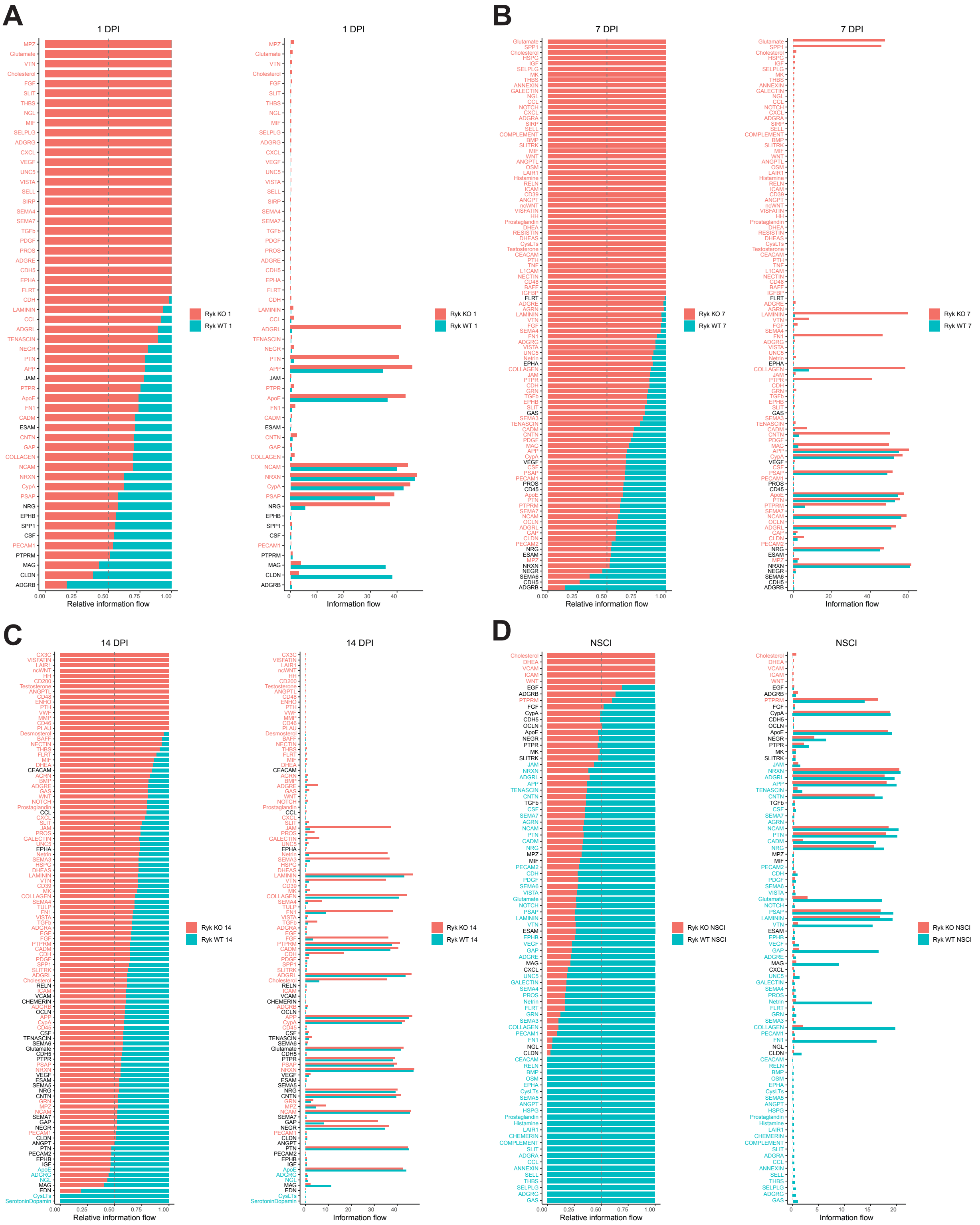

A
1 DPI
1 DPI
Relative information flow
Information flow
Ryk KO 1
Ryk WT 1
B
7 DPI
7 DPI
Relative information flow
Information flow
Ryk KO 7
Ryk WT 7
C
14 DPI
14 DPI
Relative information flow
Information flow
Ryk KO 14
Ryk WT 14
D
NSCI
NSCI
Relative information flow
Information flow
Ryk KO NSCI
Ryk WT NSCI

Sup. Fig. 13

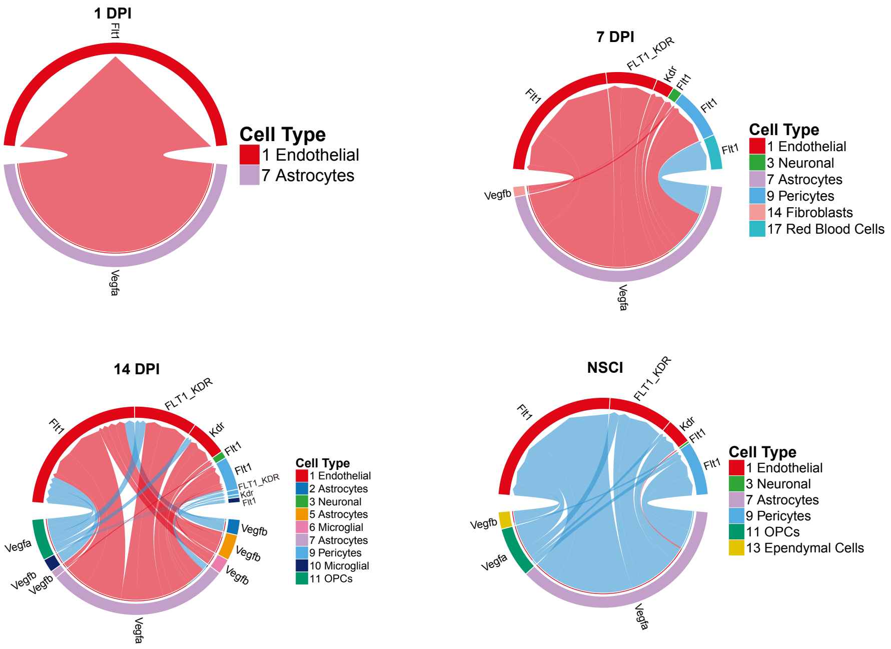

1 DPI
Flt1
Vegfa
Cell Type
1 Endothelial
7 Astrocytes
7 DPI
Flt1
FLT1_KDR
Kdr
Flt1
Flt1
Flt1
Vegfb
Vegfa
Cell Type
1 Endothelial
3 Neuronal
7 Astrocytes
9 Pericytes
14 Fibroblasts
17 Red Blood Cells
14 DPI
Flt1
FLT1_KDR
Kdr
Flt1
Flt1
FLT1_KDR
Kdr
Flt1
Vegfb
Vegfb
Vegfb
Vegfa
Vegfa
Vegfb
Vegfb
Cell Type
1 Endothelial
2 Astrocytes
3 Neuronal
5 Astrocytes
6 Microglial
7 Astrocytes
9 Pericytes
10 Microglial
11 OPCs
NSCI
Flt1
FLT1_KDR
Kdr
Flt1
Flt1
Vegfb
Vegfa
Vegfa
Cell Type
1 Endothelial
3 Neuronal
7 Astrocytes
9 Pericytes
11 OPCs
13 Ependymal Cells

Sup Fig 14.

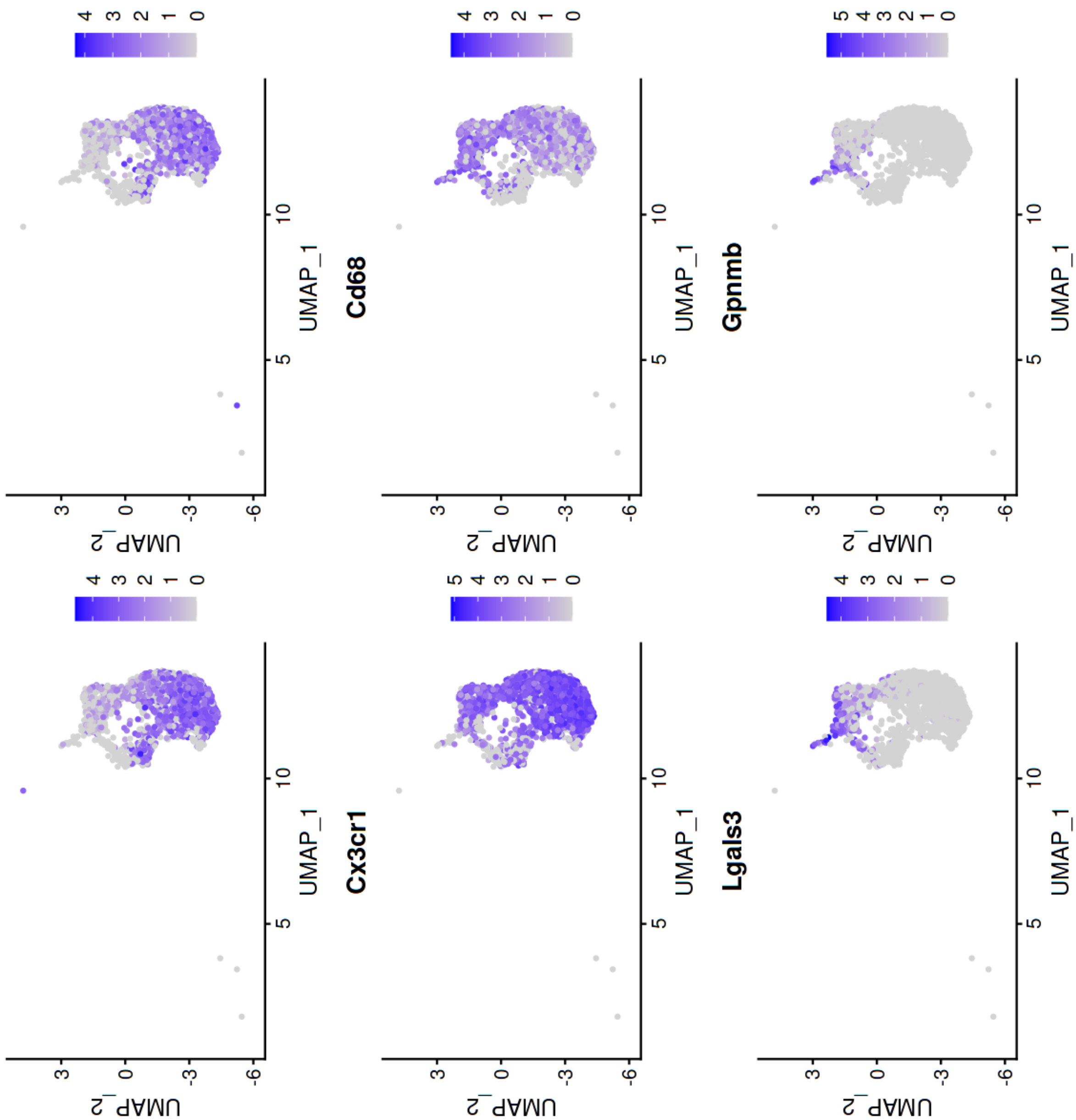

Sup Fig 15.

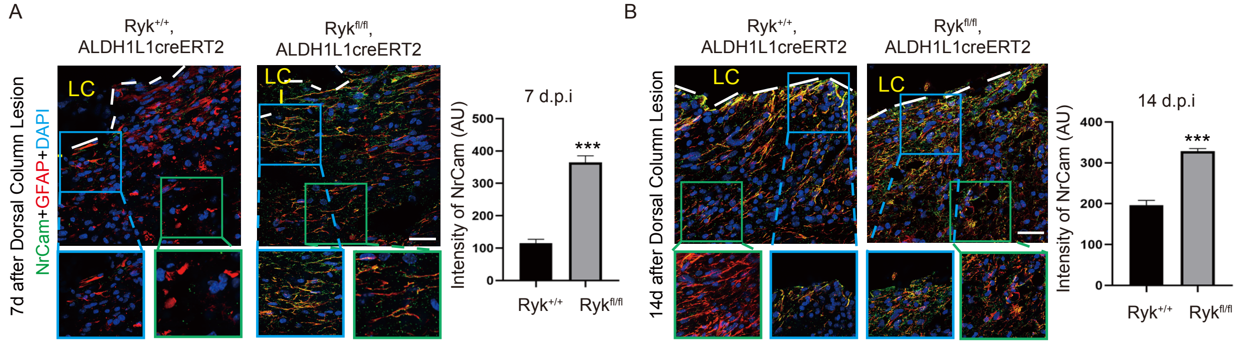
A
Ryk+/+, ALDH1L1creERT2
Rykfl/fl, ALDH1L1creERT2
7d after Dorsal Column Lesion
NrCam+GFAP+DAPI
LC
7 d.p.i
Intensity of NrCam (AU)
500
400
300
200
100
0
***
Ryk+/+
Rykfl/fl
B
Ryk+/+, ALDH1L1creERT2
Rykfl/fl, ALDH1L1creERT2
14d after Dorsal Column Lesion
LC
14 d.p.i
Intensity of NrCam (AU)
400
300
200
100
0
***
Ryk+/+
Rykfl/fl

Sup Fig 16.

A

NrCam antisense oligo (ASO)

Control: G*C*G*A*C*T*A*T*A*C*G*C*G*C*A*A*T*A*T*G
NrCam-1: C*C*A*C*G*C*T*G*A*C*G*C*G*A*A*C*A*T*T*T
NrCam-2: C*T*G*T*C*G*T*G*C*G*T*G*T*T*T*C*C*G*A*A
NrCam-3: G*A*C*G*G*C*T*C*C*T*A*A*T*G*C*G*T*T*T*T

B

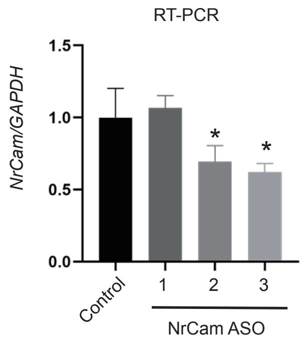

Supplement: Supplementary file 1 — Appendix 01 (PDF) [file pnas.2417400122.sapp.pdf]
